# A new heuristic framework for estimating indirect (Scope 3) emissions of large organizations

**DOI:** 10.1038/s41598-025-17902-5

**Published:** 2025-10-21

**Authors:** Viraj Sawant, Kaiyi Wang, Vicky Tong, Zachary Fendler, Bala Krishnamoorthy, Seema Gandhi, Deepak Rajagopal

**Affiliations:** 1 Institute of Environment and Sustainability, University of California, Los Angeles, USA; 2 Department of Anesthesia and Perioperative Care, University of California, San Francisco, USA; 3 Department of Mathematics and Statistics, Washington State University, Vancouver, USA

**Keywords:** Environmental impact, Climate-change mitigation, Sustainability

## Abstract

Large businesses and organizations are pledging voluntary actions for climate change mitigation. Alongside such pledges, government regulations that mandate climate-related disclosures for large corporations are also emerging. Both for compliance with such mandates and effective voluntary action, organizations need reliable information on their indirect emissions, which refers to emissions arising upstream and downstream to their own operations, and which, for many types of industries (such as education, finance, health care, hospitality and retail) can be several-times their own or direct emissions. However, the best approach to estimating indirect emissions requires conducting a life cycle assessment (LCA) for a large number (potentially thousands) of different products and services, which is costly. To overcome this, we present a heuristic approach that combines insights derived from LCA and data analytic techniques to identify a relatively small number of products that might account for a large share of indirect emissions, specifically Scope 3 emissions. We apply our approach to a dataset comprising over 25,000 products spanning 105 different product categories, purchased by a large tertiary-care hospital and show that this can help organizations prioritize actions aimed at reducing their indirect emissions.

**Supplementary Information:**

The online version contains supplementary material available at 10.1038/s41598-025-17902-5.

## Significance statement

For many different types of industries, the so-called Scope 3 emissions can account for a substantial share of their overall environmental footprint. Today the widely-used approach to estimating scope 3 emissions involves relying only on information about unit cost of the various products and activities and an average life cycle emissions per dollar for the closest broad industry-sector that each product or activity can be assigned to. The alternative heuristic developed here harnesses both information on cost and physical quantity, which suggests prioritizing a very different set of products for reduction in Scope 3 emissions.

**Supplementary Information:**

The online version contains supplementary material available at 10.1038/s41598-025-17902-5.

## Introduction

Large businesses, both for-profit corporations and not-for-profit organizations, are pledging voluntary carbon mitigation. Alongside such pledges, regulations such as the European Union’s Corporate Sustainability Reporting (CSR) Directive (2022), California’s ‘Climate Corporate Data Accountability Act (2023), U.S. SEC climate-disclosure rules (2024) are advancing mandatory climate-related reporting for large companies^1–3^. For both voluntary action and regulatory compliance, organizations need detailed information about their lifecycle environmental footprint. The lifecycle footprint of a product or an organization can be classified into direct and indirect emissions. Direct emissions are those arising from an organization’s own properties (facilities and vehicles), while indirect emissions are those associated with upstream and downstream activities that enable and support the organization’s own activities. For many industries such as education, finance, health care, hospitality and retail, indirect emissions can be several-times their r direct emissions. An alternative classification popular in corporate sustainability reporting CSR-context is Scope 1, 2, and 3 emissions as described by the GHG Protocol^4^. Scope 1 emissions are simply the direct emissions. Scope 2 emissions refer to a subset of indirect emissions that are associated only with electricity, steam, heating, and cooling services purchased by an organization for use in its own facilities. Scope 3 emissions are the rest of the indirect emissions, which include lifecycle emissions from all other upstream and downstream activities. The GHG protocol divides these Scope 3 activities in 15 distinct categories that includes purchased goods and services, transportation, employee commuting, business travel, use and end-of-life management of sold products, and waste management, among others^5^. Here, we present a case study of indirect emissions in healthcare systems, specifically focusing on one category, ‘Purchased Goods and Services’, of the Scope 3 emissions as defined by the GHG Protocol.

The healthcare industry is estimated to be responsible for more than 4% of the global greenhouse gas (GHG) emissions and 8.5% of the domestic GHG emissions in the United States^6–8^. With increasing median income and an aging population, demand for health services is growing rapidly globally^9–11^. This has driven a growing interest in reducing Healthcare industry’s increasing contribution to climate change^6,12–17^ In 2022, the White House and the Human and Health Services (HHS) launched the Health Sector Climate Pledge to encourage the healthcare sector’s commitment and actions in decarbonization. This pledge includes conducting an inventory of Scope 3 supply chain emissions, a task many health systems have never undertaken before. Most voluntary disclosures and pledges thus far have focused on Scope 1 and 2 emissions^18–20^.

Although scope 3 emissions are relatively more difficult to estimate, which motivates this work, the state-of-the-art technique (described below) suggests they might be several-fold greater than Scope 1 and 2 emissions combined^21^. For instance, Eckelman et al. (2020) estimate that Scope 3 indirect emissions account for 82% of the total US healthcare emissions, while a study undertaken for the University of California Office of the President concluded Scope 3 indirect emissions contributed 72.5% of the total emissions among the five academic health systems in 2022. A widely-used approach, which in this study we will refer to as ‘top-down approach’, proceeds as follows for purchased goods emissions: (i) Collect expenditure data on different products and activities relevant to scope 3 emissions; (ii) Match each product or activity with one of the 400 or more broad industry groups listed in the environmentally-extended input-output (EEIO) tables; (iii) Multiply the expenditure on each individual product and the average GHG emission intensity per dollar of output for the matched g EEIO sector to estimate lifecycle emissions due to that product; (iv) Sum the emissions of each product to determine total scope 3 emissions for the organization^22^. This approach is also called the ‘expenditure-based’ method in the literature^20^. The main limitations of using a purely expenditure-based approach to estimate emissions are twofold. One is that it lacks the granularity to adequately accommodate the diversity of the products and services and their lifecycle emissions^23^. For instance, for the healthcare industry, which we use as a case study here, a single large hospital purchases over 25000 distinct products (grouped under 105 product categories), which are matched to only a handful of the EEIO categories. The second is that both the cost of production and the retail price of a product are poorly correlated with environmental footprint^24^ (See Fig S1). Therefore, there is a need for better approaches to identify the most polluting products and prioritize mitigation strategies.

The ideal approach, which we refer to as a ‘bottom-up approach’, is to conduct a life cycle assessment (LCA) for each product or activity contributing to scope 3 emissions^25^. An LCA is a standardized methodology (ISO 14040, ISO 14044) to estimate the different types of environmental and resource burdens associated with the entire life cycle of a product or service. The GHG protocol defines this approach as ‘supplier-specific method’ where a business has to “collect product-level cradle-to-gate GHG inventory data from goods or services suppliers”^5^. There is growing literature on LCA of healthcare-related products and services. Examples include personal protective apparel (PPE) such as scrubs and head covers^26–30^, face masks^31–33^, gloves^34^, and specific medical instruments and devices, e.g., anesthetic equipment^35^, laryngoscope^36^, ureteroscope^37^, catheter^38^, and knee implants^39^. Going beyond individual products, literature also analyzes different medical procedures that are multi-product systems, e.g., prostatectomy^40^, and cataract surgery^41,42^. A limited but growing literature analyzes emissions for specialized care, such as for bariatric surgery^43^, hemodialysis treatment^44^, dental care^45,46^, and intensive care^47^. Drew et al. (2021) reviewed the landscape of LCA studies related to surgical and anesthetic care^48^. However, the number of peer-reviewed, published and publicly accessible LCAs is but a tiny fraction of the tens of thousands of products consumed in hospitals, and more generally, in the healthcare sector. But since conducting an LCA for each product is data intensive and costly in time and resources, a purely bottom-up approach to Scope 3 emissions appears impractical. An LCA of each products is perhaps even unnecessary given the distribution of products in terms of the quantity of and expenditure on each (more details below).

The few hospital-level studies that exist apply either a top-down approach or a hybrid approach, where some products are subjected to a bottom-up method, with the rest subjected to the expenditure-based method. A hybrid approach used in a study of a German hospital includes the bottom-up method for Scope 1 and 2, but the top-down method for Scope 3 emissions^25^. A study of a Dutch hospital finds the annual carbon footprint to be 209 kilotons, with Scope 3 contributing the majority share at 72%^49^. One study that employs the bottom-up approach exclusively is an Organizational LCA of a Canadian hospital^23^. Organizational LCA (O-LCA) is a framework that expands LCA methodology for an organization where the functional unit is a financial reporting year^50^. O-LCA methodology applied to the relevant products can be used for calculating the Scope 3 emissions of the organization for that reporting year. The challenge with this approach is selecting a representative sample from the large number of products consumed in the organization to best approximate total emissions or identifying the major contributing products. The O-LCA guidance does not mandate any specific sampling approach except recommending a few alternatives and requiring transparent communication of the choices made. Cimprich and Young (2023) adopt a stratified random sampling method to analyze a portfolio of 2927 unique products, where the sample size is determined by the LCAs of pilot products in different categories. They compile life cycle inventory (LCI) data for a sample of around 200 products based on existing literature and primary data collection^23^.

Here, we present a different heuristic framework for estimating scope 3 emissions and apply it to data from the University of California, San Francisco Medical Centre, a tertiary academic medical center with a total of 796 beds across its various facilities. We specifically analyze one category of scope 3 emissions called purchased products, which is currently estimated to constitute the biggest part of the Scope 3 emissions. Our dataset comprises over 25,000 unique medical products categorized into 105 different categories and amounting to over $290 million in annual expenditure by UCSF. Given the large number of products, the lack of readily available estimates of lifecycle emissions for most of these products and the challenges in performing an LCA for each, we develop a heuristic technique to identify a subset of all products for each of which we develop an estimation of life cycle emissions which we then scale to estimate emissions for all the products in our dataset.

**Fig. 1:**
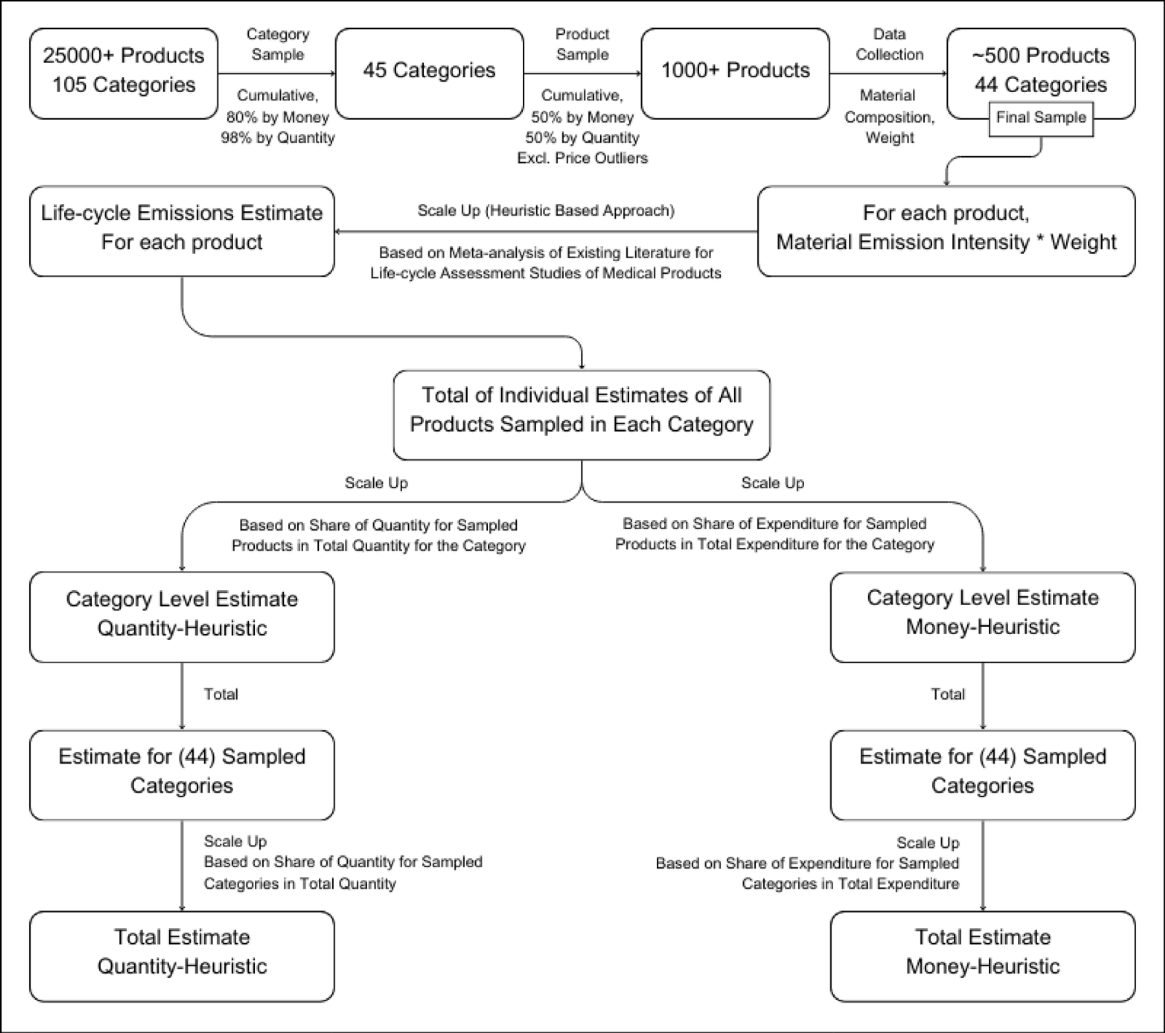
Schematic of the proposed bottom-up approach in this paper.

We present a heuristic to obtain a sample of ~ 1000 products spanning 45 categories from a population of over 25,000 products spanning 105 categories. Our heuristic uses information about both the expenditure on and quantity of different products. Next, we estimate lifecycle emissions for each of these products using another heuristic. From this, we calculate emissions for each category. We do this by totaling the emissions for all products in the sample from a given category and then scaling this by the inverse of the ratio of total expenditure on all products in that category to the total expenditure on the products in the category that are in the sample. In this way, we calculate the total emissions across the sampled categories. We then scale this by the inverse of the ratio of total expenditure across all categories (i.e., 105 categories) to the total expenditure on the categories in the sample (See Method for more details and Fig. 1 for a schematic of our approach). The estimate of total emission derived this way is what we refer to as the “*bottom-up money heuristic”.* Using the same two-step process, we also derive a “*bottom-up quantity heuristic”* wherein the scaling at the first step is done based on the share of total quantity that products in the sample represent for a category and then again in the second step, scaling is done based on the share of total quantity across all categories represented by the categories in the sample (see Fig. 1 for schematic of our approach).

The main objective of this work is to present an alternative to the expenditure-based heuristic and show how this suggests a different set of priorities for emission reduction. Indeed, one could improve our heuristic or develop alternative heuristics, and for this reason, we don’t claim ours is optimal, but as one that does not rely purely on cost and average emissions per unit cost for highly-aggregated economic sectors, as the expenditure-based method does. Indeed, our heuristic does entail arbitrary cut-offs such as the share of expenditure and quantity yet these are based on plausible justifications. To address this, we perform a sensitivity analysis. Additionally, we develop a metric of uncertainty using machine learning techniques (natural language processing using neural networks and unsupervised learning) to analyze text descriptions of the products. This metric is a measure of how aligned the sample for each category is with the product diversity within that category. We believe that the heuristic approach presented here, combined with an understanding of sensitivity and uncertainty, holds promise for identifying cost-effective interventions for reducing scope 3 emissions.

## Results

**Fig. 2:**
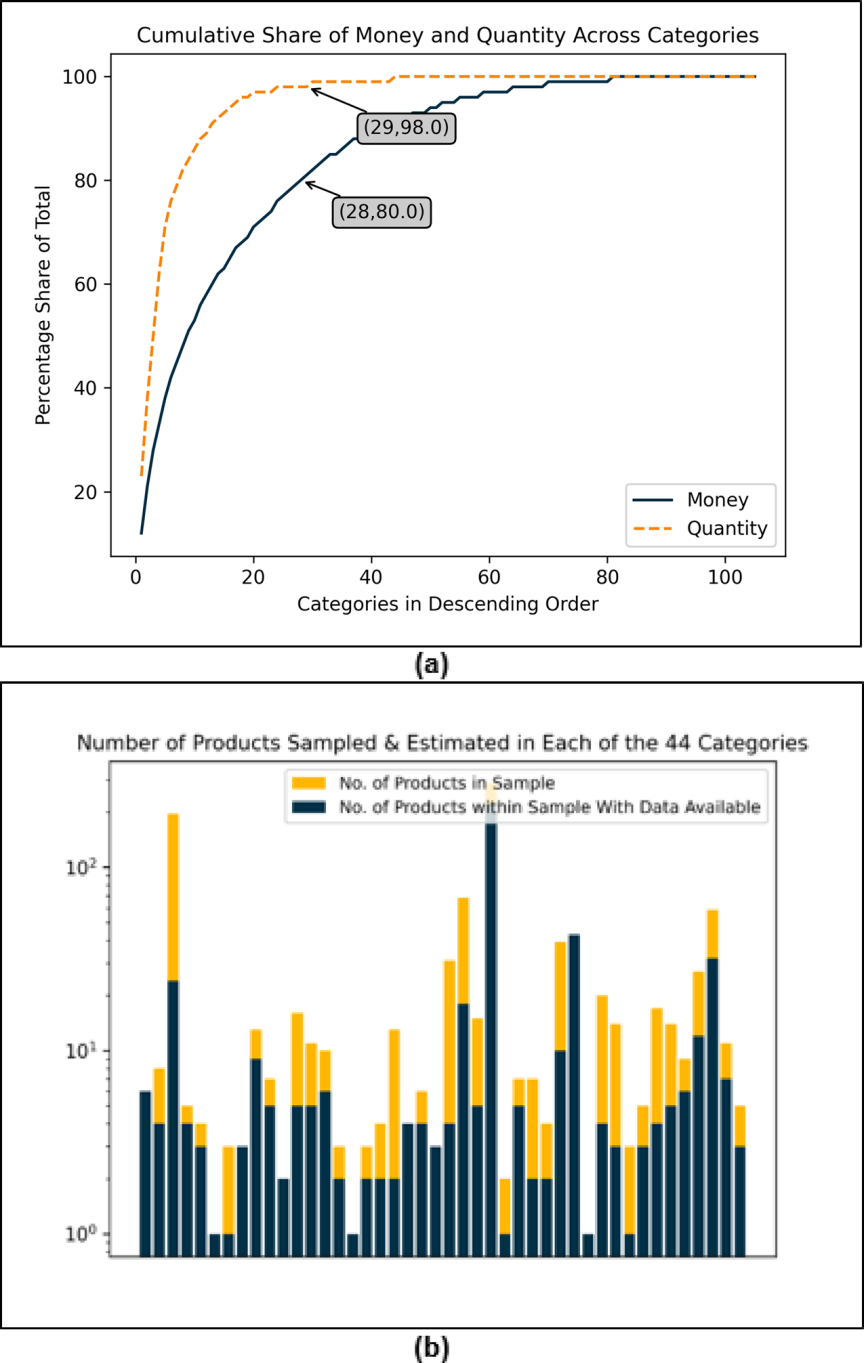
(**a**) Cumulative shares of quantity and expenditure in different categories sorted in descending order, (**b**) Each bar represents one of the 45 categories sampled with a number of products in the sample and the number of products where data needed to estimate the emissions were available. The total number of products with available data across all the categories is 492.

The distribution of both quantity and expenditure at a product category-level shows that a small proportion of categories account for most of the consumption (Fig. 2a). Of the total 105 categories, top 29 account for 98% of all total quantity consumed while top 28 categories account for 80% of total expenditure. To limit the number of distinct product categories for which information on lifecycle impacts would need to be gathered or estimated, we use these two arbitrary thresholds of 80% of total expenditure and 98% of total quantity of products, the union of which yields 45 distinct categories. For each category in this 45-category subset, we next select a subset of products within each category for which we estimate a product-specific carbon footprint per unit quantity (more detail under Methods). Looking again at the cumulative distribution by both expenditure and quantity within each category in the subset of categories, we observe again that a relatively small share of products accounts for a large share by both expenditure and the number of units purchased within each category (See Fig S5 and S6). Again, to limit the number of distinct products chosen from within each category for further analysis, we applied an arbitrary threshold of 50% of total expenditure and 50% of total quantity within each category (more detail under Methods). These two steps, selection of categories and selection of products with each selected category, yields a total of 1018 distinct products spanning 45 distinct categories for each of which we estimate lifecycle GHG emissions. Figure 2B shows distribution of number of selected products across the selected categories. Since essential data on material composition and products weights for lifecycle estimation of each product was available only for a limited number of selected products, our final sample consists of 492 products across 44 categories for which we were able to estimate lifecycle emissions.

**Fig. 3:**
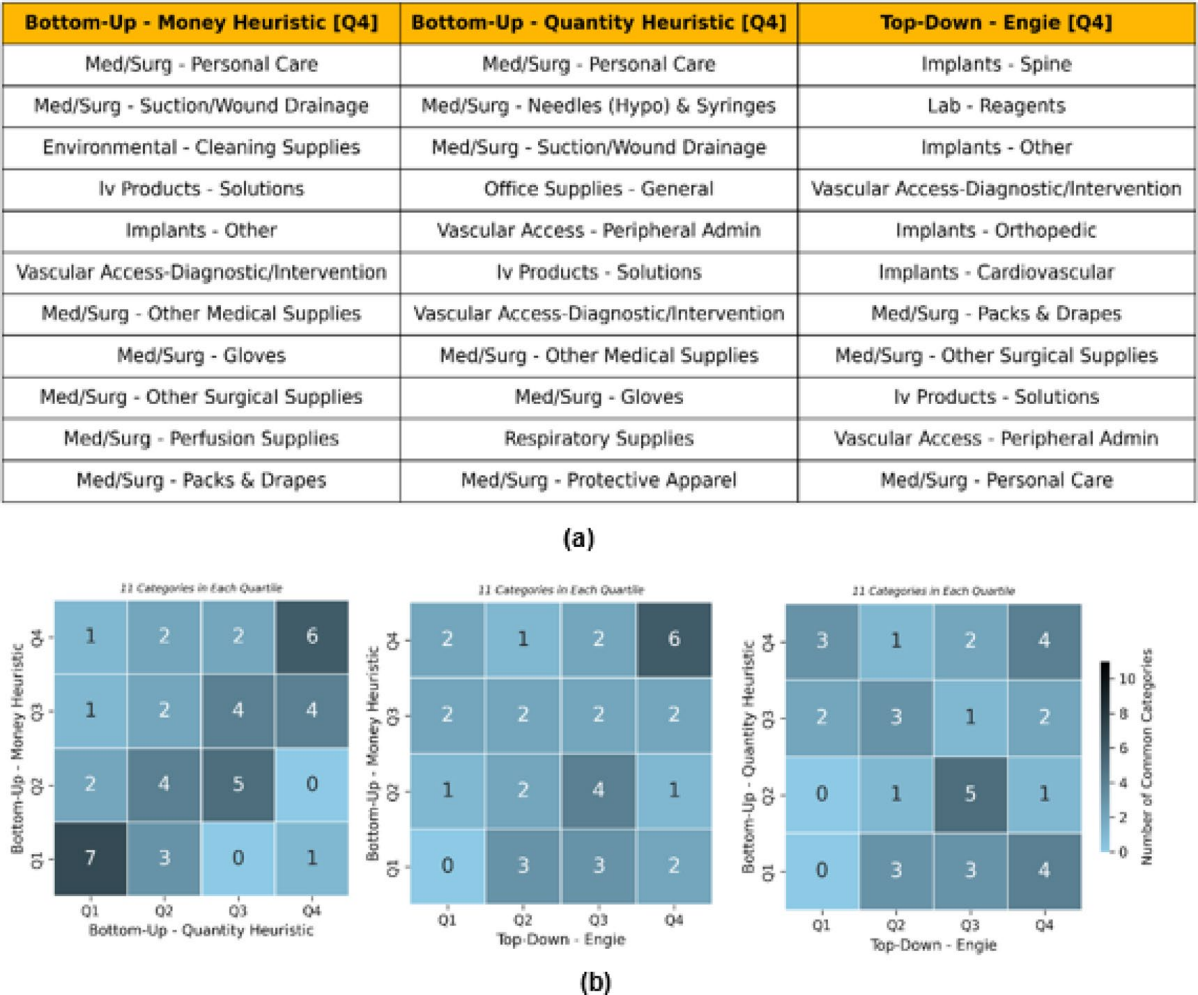
(**a**) Top quartiles of categories by their contribution to the total emissions as per three different methodological approaches. (**b**) Pair-wise comparison of the three approaches. The values in squares represent number of product categories common in the corresponding quartiles of the two criteria being compared. With a total for 44 categories, each quartile consists of 11 categories.

Table 1 shows estimates derived using our two heuristics - bottom-up money and bottom-up quantity and an estimate using the top-down approach (see Methods for detail). While each of the three approaches yields estimates that are similar in magnitude, both our heuristics yield substantially lower estimates compared to that from a purely expenditure-based approach. But more importantly, each approach yields a different ranking of the specific categories of products (as well as the ranking of products within each category) that are estimated to contribute the most emissions. Most notably, whereas the top-down approach identifies four different types of implants related product categories among the top contributors (top quartile), only one of our two heuristics identify a single type of implant in the top contributors (Fig. 3a). Since implants are amongst the costliest products in our dataset, the top-down expenditure-based approach is more likely to identify them as an important contributor to total emissions even though in reality they may be less pollution-intensive than many cheaper and widely used products.

**Table 1:** Total emissions estimate for the product dataset as per different methodological approaches.

Methodological approach	Estimate (Tonnes CO2)	Description
Bottom-Up [Money Heuristic]	~ 64,000	Emissions estimate for the product sample in each of the 44 categories is scaled up using the share of sample in total money spent in the respective category and then total emissions for 105 categories is obtained from scaling up the total emissions for 44 categories using their share (~ 80%) in total money spent.
Bottom-Up [Quantity Heuristic]	~ 44,000	Emissions estimate for the product sample in each of the 44 categories is scaled up using the share of sample in total quantity purchased in the respective category and then total emissions for 105 categories is obtained from scaling up the total emissions for 44 categories using their share (~ 98%) in total quantity purchased.
Top-Down [Expenditure Based-Heuristic]	~ 84,000	Each of the 105 product categories in the dataset are matched to the appropriate categories in the *Engie* tool which is based on the US EPA’s Environmentally Extended Input Output (EEIO) tables. The expenditure on each category of products is multiplied by the emissions per dollar for the corresponding EEIO category to estimate category level emissions which is then aggregated across all product categories.

We also did a pair-wise comparison of the three approaches – our two heuristics-based bottom-up approaches and top-down approach and analyzed the extent of overlap for each quartile of the 44 categories (Fig. 3b). A greater share of common categories in the same quartiles as per different approaches indicates greater agreement between them. As depicted in Fig. 3b, larger numbers along the diagonal suggest greater overlap between the two approaches, while larger numbers in off-diagonal cells suggest the categories are distributed in different quartiles as per different approaches. Note that for the figure showing our two bottom-up approaches, each row, and each column will add up to 11 because both heuristics are applied to the same set of 44 categories. On the other hand, the set of the top 44 categories selected as per the top-down technique is not identical to the set of 45 categories identified by our approach. We find that the two bottom-up approaches appear to be in greater agreement with each other than with the top-down technique, as indicated by larger off-diagonal values.

We can also see that the bottom-up money heuristic appears to overlap more with the top-down approach as compared to the overlap of the bottom-up quantity heuristic with the top-down. The agreement is stronger in Q4 (with 6 of categories being the same for top quartiles as per the two approaches). The bottom-up quantity heuristic has much less in common with the top-down approach with no categories common in Q1s and only 1 common in Q2s and Q3s of both approaches with the highest common value of 4 observed in Q4s.

Building on an earlier example, we again see that high-priced products are where the most difference is seen in the rankings. The product category ‘Implants – Other’, which is in the top quartile as per the money heuristic approach, is in the last quartile as per the quantity heuristic. The top category identified by both bottom-up approaches - ‘Med/Surg - Personal Care’ - includes various types of cleansing clothes, wipes, cleaning solutions (e.g., alcohol), and miscellaneous products related to patient hygiene, among other products. These are typically low-priced items that expectedly rank lower when analyzed using the top-down approach at 11 (out of 44). The top-down approach ranks categories with high-priced products like implants with ‘Implants – Spine’ at the top of the chart. The bottom-up approach ranks these categories significantly lower than others (Tables S1, S2, S3). These disagreements in results coming from different methodological approaches are consequential when planning sustainability strategies for the hospitals. Even though the total emissions reported may not be affected significantly, the choice of approach will have an impact on the prioritization of product categories for emission reductions.

### Sensitivity analysis

Since there do not exist publicly reported estimates of lifecycle emissions for most products in our sub-sample (and the full set) and since conducting an LCA for each of the hundreds of selected products is beyond the scope of our research, we developed another heuristic. For each product in our sample, we identified the primary material constituting the product. In this way we identified different types of plastics, stainless steel, natural fiber, titanium as common key materials across various products. Next, we reviewed the literature on LCA of different products, both medical and non-medical, made with each of these specific materials and derived a mean estimate for the share of emissions that primary material manufacturing accounts for in total emissions associated with final products made with that material. We then use this mean along with the weight share of the primary material in the final product in our sample to estimate lifecycle emissions per unit product. More detail is provided in the materials and methods section.

Coincidentally, employing this approach yields an estimate of aggregate emissions that is in the same order of magnitude as the top-down approach. Of course, as the share of material emission intensity in total lifecycle estimates changes, this will affect our estimate of total emissions. In Figure S2, we demonstrate this by applying different scaling factor values uniformly across all products, irrespective of the material. If the share of material emission intensity in total lifecycle emissions is lower, i.e., contributions from other areas like processing and transportation are higher, the emissions estimates increase rapidly. This sensitivity is important to consider as there may be significant variations in the supply chain that could impact the actual lifecycle emissions of products.

**Fig. 4:**
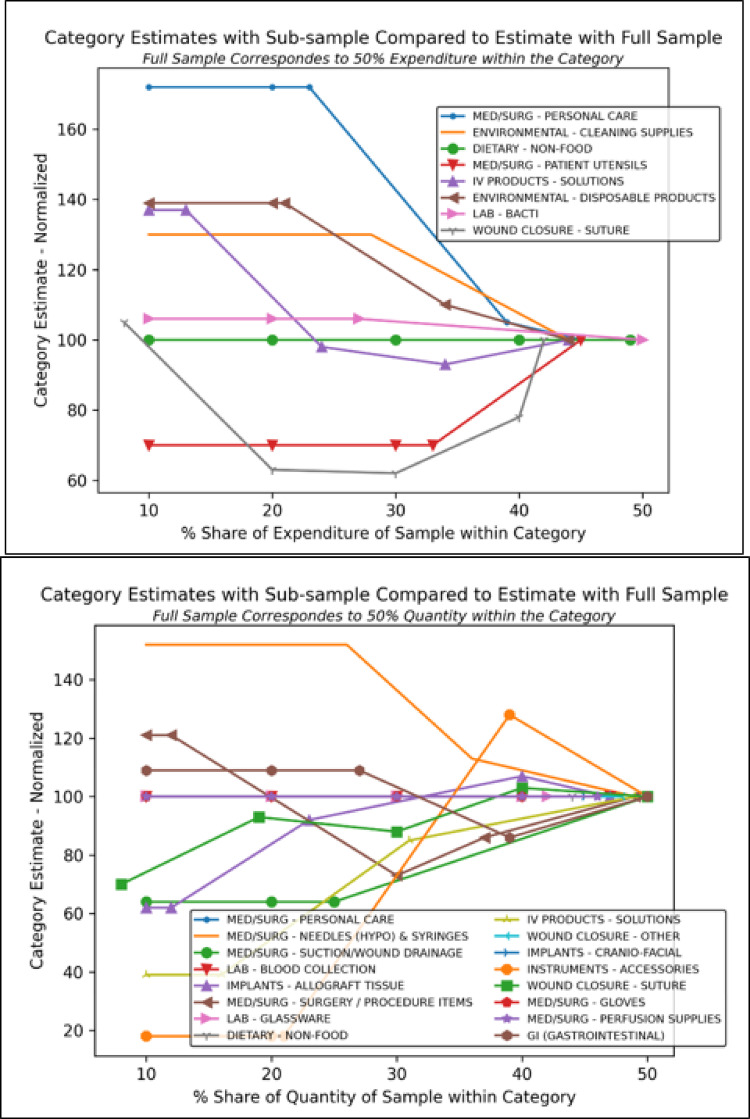
Change in the emission estimates for different categories change with sample size. The estimates for subsamples are normalized to the estimate with the full sample. We show here only categories where full sample corresponds to ~ 50% level after the data collection.

Figure 4 shows the sensitivity of category level estimates to the threshold of 50% set for both heuristics in each category to select a sample of products. The figure shows plots for those categories for which we obtained data on weights and material composition for around 50% of the products within the category by either expenditure or quantity. Emission estimate with a 50% sample is considered as a reference – 100% - and estimates obtained using smaller samples are normalized to the reference. Only few categories show a small difference between estimates with smaller samples and the reference – e.g., ‘Med/Surg - Gloves’. A small difference implies that one could obtain robust estimates with a smaller sample in that category, thus reducing the burden of analyzing a larger number of products. This is supported by the fact that the product category containing gloves appears more homogenous in the different specific products it contains when compared to other categories. Most other categories exhibit greater sensitivity when using smaller samples. A few categories exhibit trends that appear to stabilize as sample size increases. For example, samples corresponding to 30% shares in categories ‘– IV Products - Solutions’ and ‘Lab - Bacti’ produce estimates marginally different from reference estimates. A greater value with a smaller sample suggests that top products are more emission-intensive and therefore a small sample would result in an overestimate of emissions of the respective product category. Likewise, a smaller value with a smaller sample would result in underestimation of aggregate emissions from that category of products.

### Product diversity and uncertainty

**Fig. 5:**
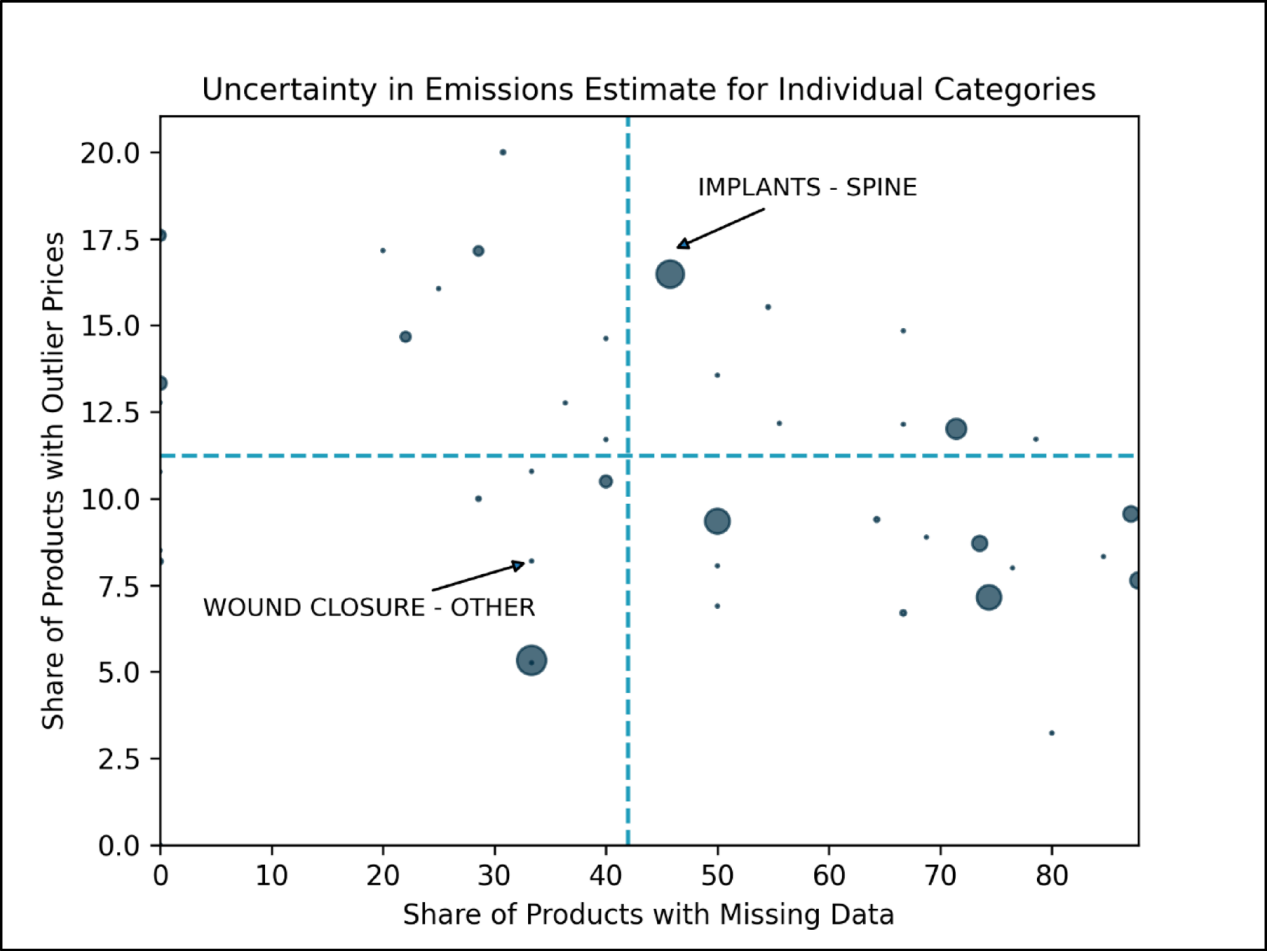
This figure contains information on three parameters: (1) share of products in a category that are classified as outliers as per unit prices, plotted on y-axis; (2) share of products in the sample where the either the material or environmental data is unavailable, plotted on the x-axis; and (3) cosine similarity between the optimum cluster distribution based on product description within a category and the representative sample of that category, the marker size is inversely proportional to negative of the cosine value. The dotted lines denote mean values for the parameters on the x- and y-axes. The higher the value of the three parameters i.e. bigger is the marker in quadrant 1, the higher is the uncertainty associated with that category.

We also analyzed the textual description provided for each product in an attempt to understand how diverse each category of products is. Using machine learning techniques to process these text descriptions of products in each of the categories we estimate the optimum number of clusters or groups of products within each category – where each cluster or a group comprises of similar products. We find that a majority of categories have a low number of distinct groups of products and are homogenous (Table S4). However, many of these homogenous categories fall outside the 44 sampled categories. We calculate the cosine similarity values, which is a measure of similarity that reduces the impact of absolute sizes of two quantities, between the sampled products within each category and their distribution in the optimal product clusters and find varying degrees of similarities. This provides a metric of confidence in our emissions estimates for the categories; the higher the similarity, the greater the ability of the product sample to represent the product diversity within that category. Figure 5 plots 44 categories for their similarity values with two other sources of uncertainty – the share of products in the category that qualifies as price outliers and the share of products within the sample where data was unavailable. Categories with high values on all three uncertainty measures need to be investigated further and we state that our confidence in estimates for those categories is lower compared to others. Therefore, in the figure, a category that is in quadrant 1 with bigger relative shares should be prioritized for further investigation through data collection and closer examination of representative samples e.g. ‘Implants - Spine’.

## Discussion

When the overall environmental footprint of an organization is spread across a large and diverse set of goods and services that it consumes and supplies both directly and indirectly, estimating the organization overall environmental footprint is extremely challenging. And without knowledge which types of goods and services contribute the most emissions, it is hard to devise a strategy for mitigating emissions. Conducting a detailed life cycle assessment of each product or service is costly and impractical. On the other hand, the current approach of relying on average emissions per unit cost of production for highly-aggregated industry sectors leads to biased estimates and result in misallocation of effort and investments aimed at pollution reduction. The heuristic framework outlined here presents a new general framework for the estimation of indirect emissions for large organizations. It combines readily available information both at product-level (cost per product, quantity consumed per product, and average lifecycle emission burden for products made of a given material) and at category level (total cost and total quantity per category, and the contribution of each category to the total expenditure) to derive a more scientifically grounded estimate of both aggregate emissions and distribution of emissions. The application to the healthcare industry suggests a different prioritization of product categories for emission reduction from the top-down approach. At a more granular level, we also show that the heterogeneity of products in categories can increase uncertainty in estimates. Recategorization - aggregation or disaggregation of products as appropriate - can be done in such a way that increases confidence in the representativeness of the product sample and thereby reduces the uncertainty in emissions calculations.

Future work can help address some limitations of this framework. One is with respect to the availability of detailed material information on specific products. In the context of LCA, this data is also known as ‘foreground data’ – the data related to the composition and manufacturing of products that allows one to build an LCA model. While intellectual property concerns are valid, even the basic information such as the weight of the unit product, and broad-level material composition information is not easily available for most products . . The other type of data needed for an LCA is ‘background data’ which includes emissions from many processes in different industries. The development of this data in the healthcare industry is still in the early stage and growth in this data through LCA studies on diverse products will allow for more accurate estimates. One avenue of future research is comparing results here with other sampling approaches and examining these results at granular level. These differences will have implications to the hospital’s ability to design mitigation strategies and effectiveness of disclosure regulations both. We also think that the machine learning pipeline developed in this study can be integrated into the sampling process. Including this in the sampling method would undermine the influence of the other two heuristics and therefore, this integration will have to be done carefully with a deeper understanding of the text descriptions of products. Growing capacities in machine learning and artificial intelligence globally make this a promising avenue to explore in future work. This can potentially lead to a more advanced framework that large organizations can adopt to estimate their indirect emissions.

## Materials and methods

The raw purchase data spans a consecutive 12 month period. Our time window does not overlap with the COVID-19 pandemic months/years which would not be representative of typical consumption. This The dataset contains 25,959 different products procured at a total cost of $293 Million USD. These products are subdivided into 105 product categories. A small share of products, about 0.6% and accounting for ~ 0.7% of the total expenditure were uncategorized and excluded from this analysis. The variables in the dataset are the manufacturer and vendor names and serial numbers, quantity purchased, expenditure, and the product category for each product.

We compare the expenditure-based approach which is widely used today with two bottom-up approaches that employ a new heuristic. Heuristics are procedures or rules to make decisions when information or time is scarce and one seeks a low cost, practical solution instead of an optimal solution^51,52^. The discussion of heuristics dates back to the notable works of the Herbert Simon (1947), and according to a review study classifies it in two main perspectives: the heuristics-and-biases paradigm, and the fast-and-frugal paradigm^53–55^. The fast-and-frugal perspective highlights benefits of heuristics in absence of information and the use of them in an adaptive way^56,57^. Heuristics are employed to deal with uncertainties; they are used to balance bias-variance dilemma where generalizability is a priority; they are used to exploit (business/market) environmental information; they are used to due to advantages of speed, transparency, and cost; and they are used to capitalize learning as their frugality allows rapid assessment, decisions-making and effective feedback mechanisms^55^.

For the bottom-up approaches, we apply two different heuristics: one for selecting a sample from the population of products and second for estimating lifecycle emissions of individual products (Fig. 1). In this study, we utilize heuristics based on existing expenditure and quantity data. This makes it generalizable across other hospitals and large organizations in general. This approach, as shown in this study, allows hospitals to make decisions about their emission mitigation strategies. The other perspective on heuristics in business literature, heuristics-and-biases paradigm, in this context, would ask a question about how close the emission estimates using this approach are to the ‘actual’ estimates. It is impossible to answer that question without conducting an LCA of each of the 25,000 + products in the dataset, which is a massive undertaking. This limitation of the study and avenue for future research has been discussed before. In this study, we analyze the differences between the emissions mitigation priorities yielded through the bottom-up approach and those through the top-down, expenditure-based approach, which is a heuristic-based approach itself utilizing expenditure and industry-average emissions data.

**Fig. 6:**
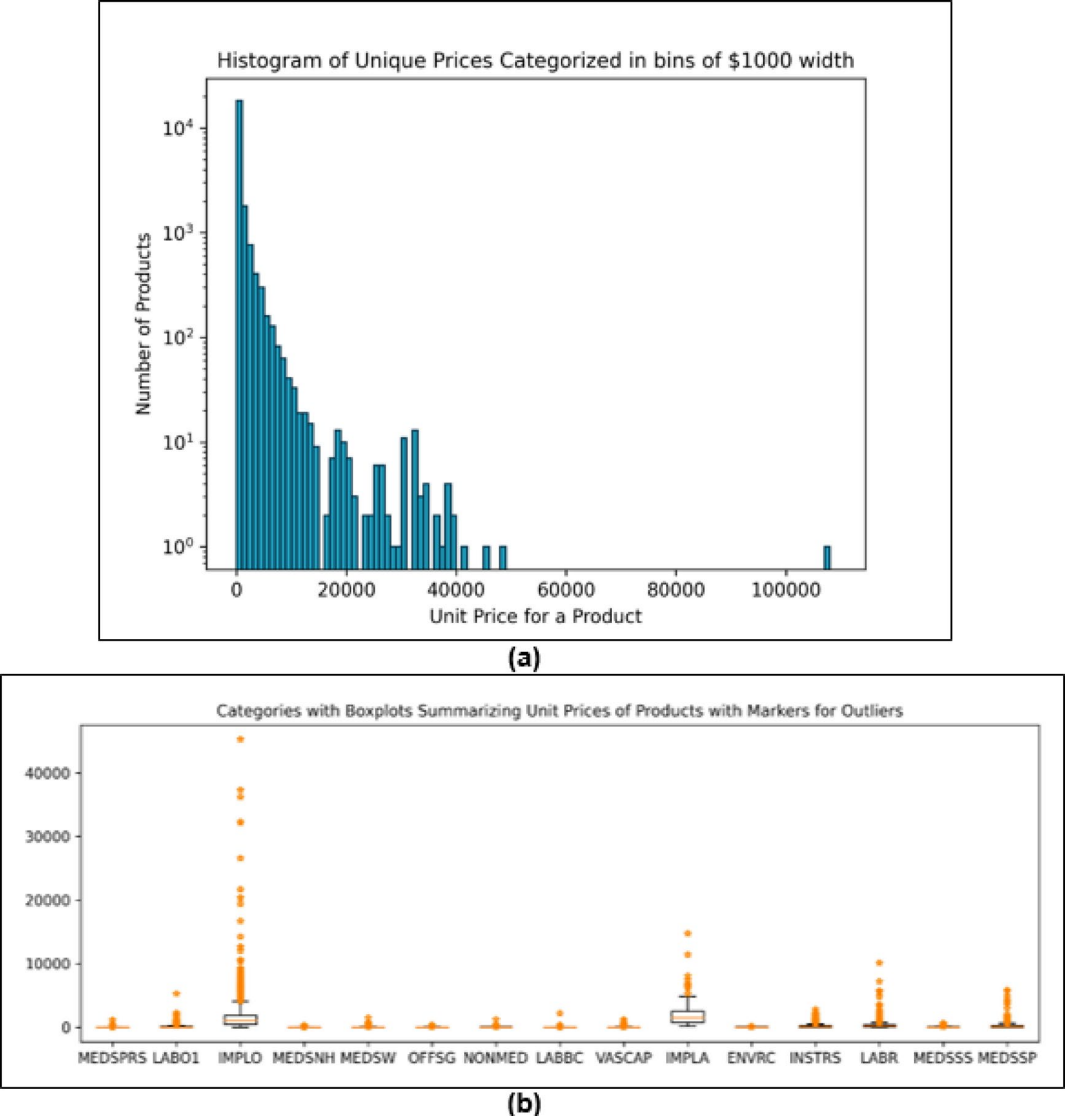
(**a**) Histogram of all individual product prices distributed in $1000 bin widths. (**b**) Boxplots and outliers of unit prices in 15 categories. Only 15 are included here for illustrative purposes. Fig S3 contains all sampled categories. Fig S4 contains further elaboration of this difference between certain categories using an example comparison.

We adopt a deterministic heuristic-based sampling approach to create a representative sample of products in two steps. In the first step, we sample the categories out of the total 105. The sampling of categories has been discussed earlier in the Results section with the help of Fig. 2. In the second step, we sample products within each sampled category. In a symmetric way to the first stage, we take a union set of top products according to both the heuristics. In this stage, the threshold is set to 50%; that is, our products in each category constitute 50% of the money spent and quantity purchased in that category. We introduce an additional filter to further shrink the sample size based on the distribution of the unit prices of products. Figure 6 shows the distribution of per unit prices of all the products in the dataset (plotted on log scale). It also shows for 15 categories the box plots of the prices of products, where an outlier is defined as any price that is outside of 1.5 times the interquartile range. As one would expect, for categories where the price distribution is tight and has few outliers, the product samples obtained from two different heuristics are quite similar whereas categories with high numbers of outliers result in much bigger product samples when both heuristics are to be accommodated (Fig. S4). Our additional filter excludes the products that qualify as outliers in their respective categories. The threshold of 50% with the additional filter results in a sample size of 1,018 products. We test multiple threshold values to explore possible sample sizes and determine 50% to be optimum based on resources available to us for further data collection and analysis (Fig. S7). We have discussed this limitation earlier.

**Fig. 7:**
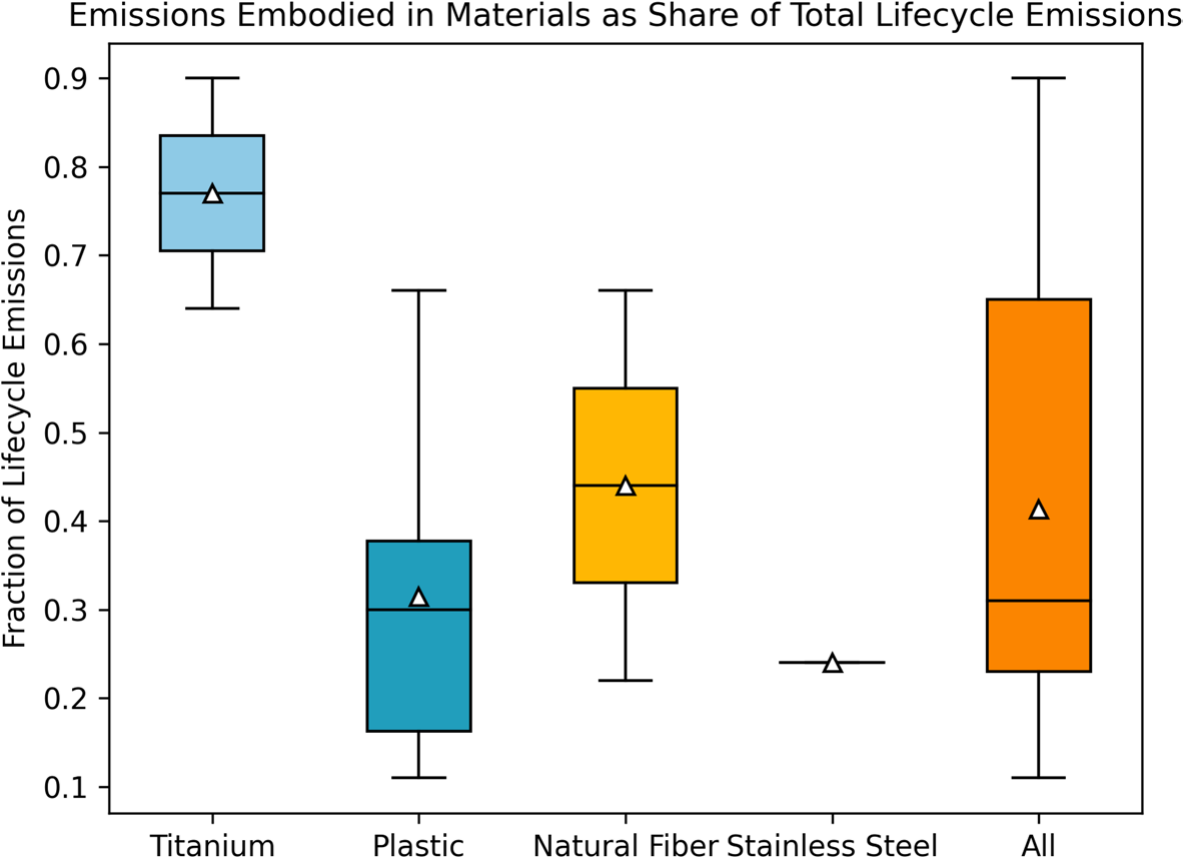
Boxplots of shares of material manufacturing in total lifecycle emissions from existing LCA studies of different healthcare system products. Different boxplots are shown for different materials.

For each product in our sample, we reviewed publicly available information on their material composition and weights. The sources for this information included vendor product catalogs, vendor websites, manufacturer product brochures, product safety declarations, and academic/medical literature connected to the product. The information about the manufacturing of medical products available in the public domain is extremely limited due to proprietary considerations. The limited product LCAs that exist in the healthcare field either procure information through partnerships with specific manufacturers or make well-educated assumptions based on public information. A hypothetical example of the latter would be a disposable plastic product that would be modeled with the weight of the specific type of plastic used and injection molding, a common manufacturing technology for plastic products. In cases where detailed information may be available for specific components and manufacturing processes, environmental data on these processes does not exist in a peer-reviewed public format. In our study, adopting such an approach to a big sample size of products would introduce a multi-fold number of parameters with a need for qualifying and analyzing the uncertainty. Instead, we use a heuristic-based approach to estimate lifecycle emissions of different products. We calculate embodied emissions in the manufacturing of the materials that make up these products and then scale it up based on the share of emissions embodied in the materials in total lifecycle emissions of medical products for which LCAs are available in the literature. We look at more than 60 LCA studies of medical products and find 11 studies where the breakdown is transparently provided, enabling us to obtain a share of total lifecycle emissions attributable to the materials themselves (Fig. 7). Once we have estimated emissions for product samples in each category, we scale them up for category-level estimates and then to the total estimate based on both heuristics separately (Fig. 1).

We developed a metric of uncertainty for our estimates using product text descriptions and machine learning techniques. The metric builds on the intuition that if a product category exhibits a greater diversity of products, then our confidence is low in the emission estimate for that category created using a sample of products. Since the text descriptions are not standardized and thus do not have an inherent structure, we employ unsupervised machine learning techniques. We use the neural network-based library Word2vec for preliminary cleaning of the text descriptions^58^. In the next step, we use the G-means cluster algorithm, a popular unsupervised learning method to identify the optimum distribution of clusters of products within each category – the clusters are made up of similar products based on their text descriptions. Once we have optimum cluster distributions, we match our sampled products against it to assess the similarity – if our sample represents clusters in optimum proportions, it gets a higher score. The score is based on cosine similarity. This creates a measure of confidence for the representativeness of the product sample that is based on another set of information that is not connected to heuristics or used in sampling.

Lastly, we also estimate emissions using the expenditure-based approach. For this, we used the ENGIE tool created by ‘Practice Greenhealth’ which is widely used in this context^59^ The ENGIE tool is, in turn, based on the Economically Extended Input Output (EEIO) developed by the United States Environmental Protection Agency (EPA), which provides estimates of average emissions intensity for each economic sector listed in the US economic input-output tables. The ENGIE tool is developed specifically for healthcare industry users to reflect common accounting practices and product/activity classifications of the industry. However, due to a lack of standardization in how hospitals record and categorize their purchases, the sectors and categories in the ENGIE tool do not align perfectly with the product categories in our dataset. We therefore had to map each product category from our dataset to the closest sector in the ENGIE tool (Table S5). This mapping, along with their expenditure, gives us category-wise emission estimates using the top-down approach, which we use later for comparison with our bottom-up approach.

## Supplementary Information

Below is the link to the electronic supplementary material.

Supplementary Material 1

## Data Availability

A portion of the data analyzed in the current study are of confidential nature specifically, data on cost and vendor for each product. However, we are open to discussing the possibility of sharing data with the approval of data provider once the article is published. Please contact the corresponding author.
