# Supplementary material for: A new heuristic framework for estimating indirect (Scope 3) emissions of large organizations
Source: Sci Rep. 2025 Oct 21;15:36539. doi: 10.1038/s41598-025-17902-5 (PMC12541089; doi:10.1038/s41598-025-17902-5)
Supplement: Supplementary file 1 — Supplementary Material 1 [file 41598_2025_17902_MOESM1_ESM.pdf]

Supporting Information for
A New Heuristic Framework for Estimating Indirect (Scope 3) Emissions of Large Organizations

This PDF file includes:

Supporting text
Figures S1 to S7
Tables S1 to S5
SI References

Supporting Information Text

Method

Preliminary Data Analysis

Our dataset consists of 25959 products and 105 unique product categories. There are some products that are uncategorized and make up a very small share, 0.71% of total expenditure. We exclude these products from our analysis as the purpose of our study is to demonstrate the methodology. Our analysis continues with 25812 products.

Machine learning based product diversity metric

Word2Vec is a popular machine learning model for generating word embeddings, which are numerical representations of words in a high-dimensional space (Mikolov et al., 2013). Word2Vec models capture semantic meanings and relationships between words based on their co-occurrences in a text corpus. These embeddings can then be used in various natural language processing (NLP) applications such as sentiment analysis, machine translation, and information retrieval. In our ML based metric, we used the Continuous Bag of Words (CBOW) architecture which takes the context of each word as the input and tries to predict the word itself. It averages or sums the context word embeddings to predict the target word, making it efficient for smaller datasets.

We used the CBOW architecture to vectorize the dataset into 200 dimensions. After creating the word embeddings, we created a copy of these embeddings and normalized the vectors. Both the normalized and non-normalized word embeddings will be used in analysis for comparison. After vectorization, we used the G-Means algorithm to perform cluster analysis on the entire vectorized dataset within their respective categories. The G-Means clustering algorithm is an extension of the K-Means clustering algorithm that automatically determines the optimal number of clusters based on the data (Hamerly & Elkan, 2004). It accomplishes this by checking if the current cluster follow a Gaussian distribution. If it does not, then the cluster may contain potential sub-clusters. The results of the G-Means cluster analysis return vectors of the cluster distribution for each category.

Given the results of the heuristic analysis, we identify which cluster they reside in their respective categories. This creates another vector which we compare to the cluster distribution using cosine similarity which is our metric for diversity. In this sense, we are checking if the selected representatives are in the right direction as the optimal distribution of clusters determined by G-Means.

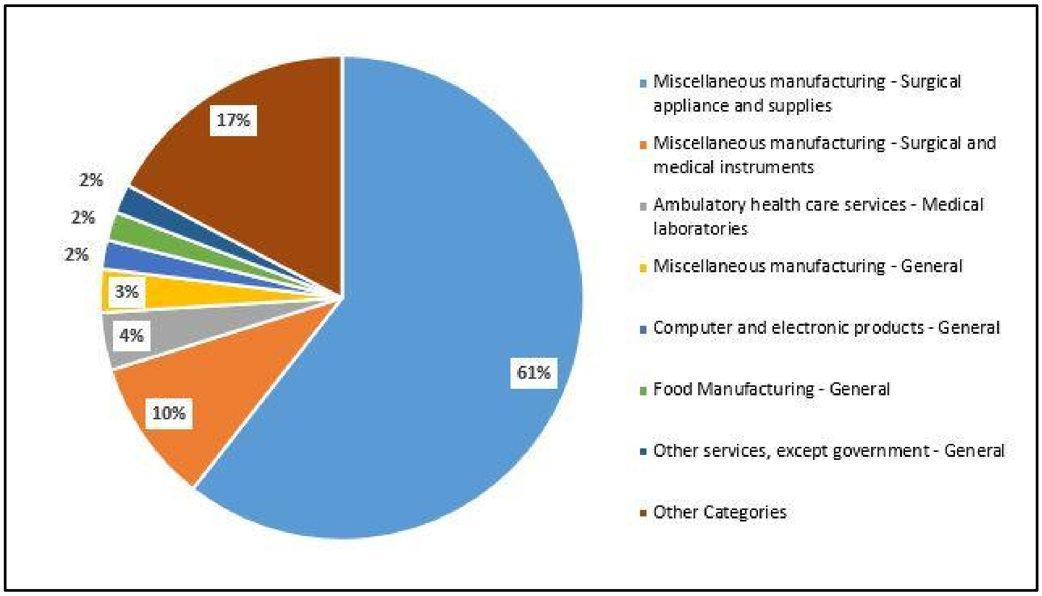

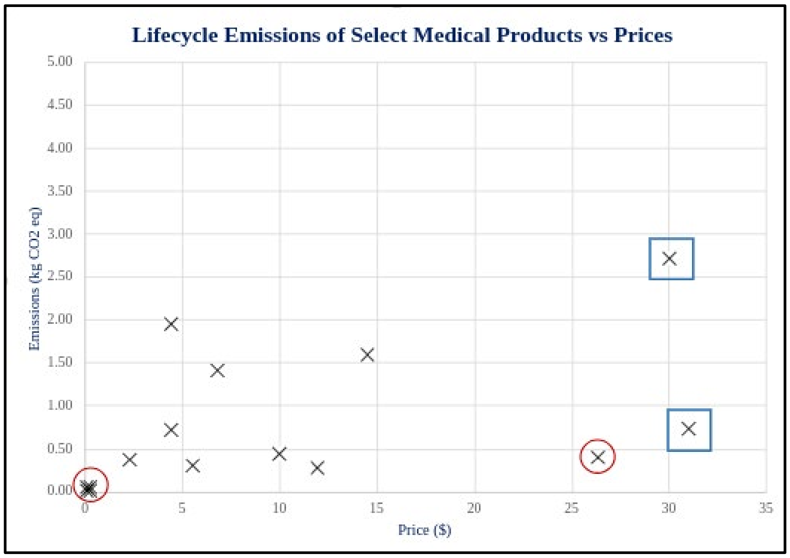

Fig. S1. (Top) This figure shows the share of 105 distinct categories in our dataset matched with Engie categories. For instance, 61% of the Hospital categories are matched with single Engie category 'Miscellaneous Manufacturing - Surgical Instruments and Supplies'. Only Engie categories which are matched with >1 Hospital categories are shown with separate slices. All other categories with 1-1 match are clubbed under 'Other Categories' which comprise only 17% of the Hospital categories. **(Bottom)** Lifecycle emissions of select products (products in UCSF dataset for which LCA data is available in literature) are plotted against their prices from hospital purchase data. The relationship between price and emissions embodied is not linear. For example, the products highlighted with two red circles have nearly the same emissions, but prices are vastly different. On the other hand, the two products highlighted with blue square marker are surgical scissors (one single use, other reusable) manufactured with different materials have nearly the same price but one has more than 3 fold the emissions of the other. With top-down approach the two scissors will be assigned the same emission/$ value, and that would lead to misleading estimates.

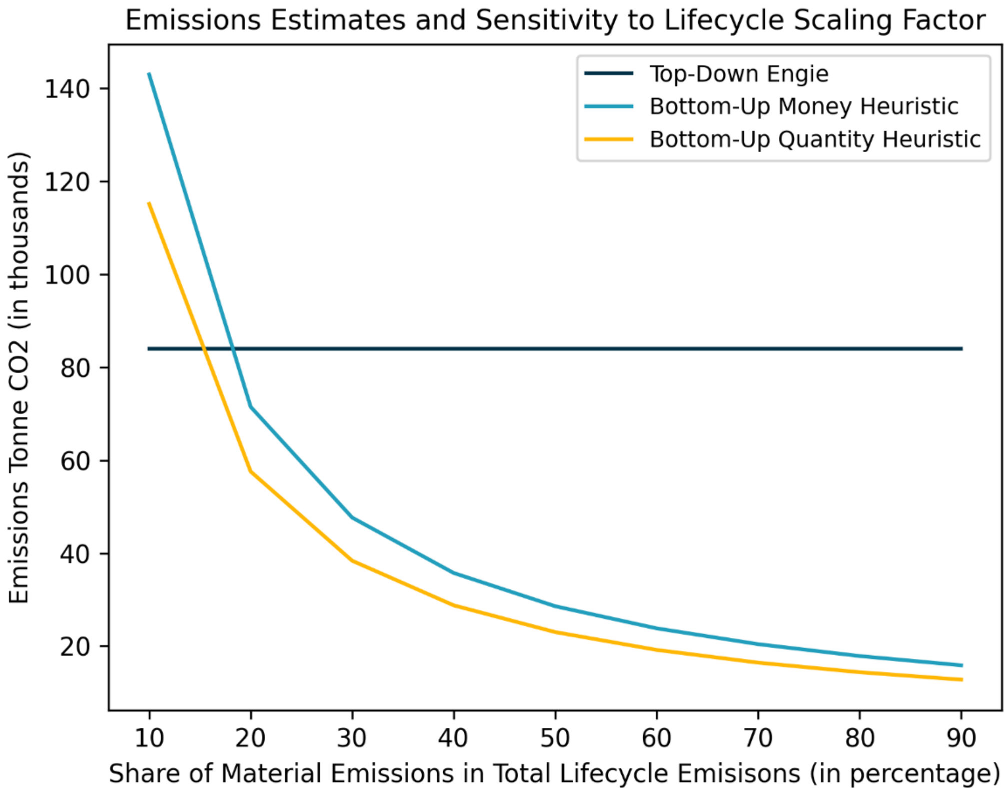

Fig. S2. Variation in estimates with changing lifeycle scaling factor. In baseline estimates we assume this factor to be 30% i.e. 30% of the lifecycle emissions of a product are from manufacturing of material of the products. This figure shows how that emissions estimate changes by changing that factor and is compared against the estimates from other approaches.

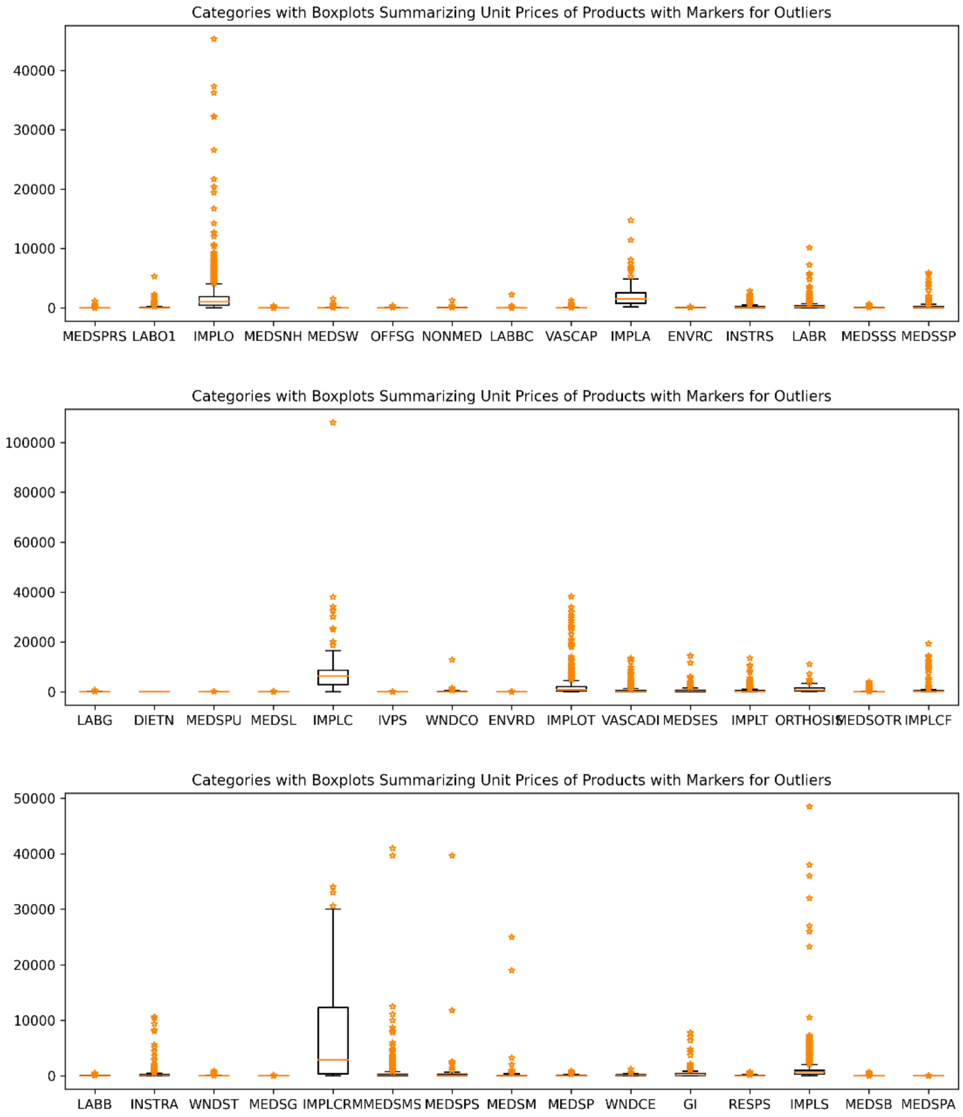

Fig. S3. Boxplots for per unit prices for all products in each of the 45 categories. Some categories show a narrow price distribution and very few outliers whereas some categories have large number of outliers.

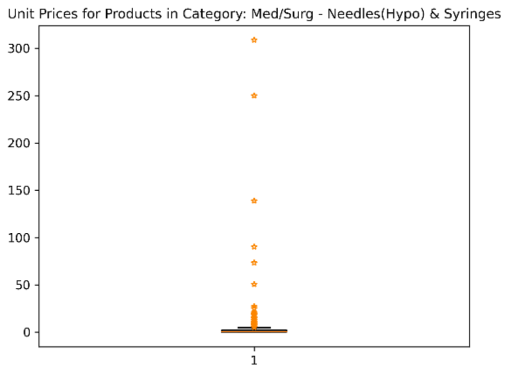

Med/Surg - Needles(Hypo) & Syringes

This category would be considered an example of a good category with a narrow distribution of per unit prices which results in largely similar rankings by quantity consumed and money spent.

count	204
mean	7
std	31
min	0.03
25%	0.15
50%	0.33
75%	1.95
max	308

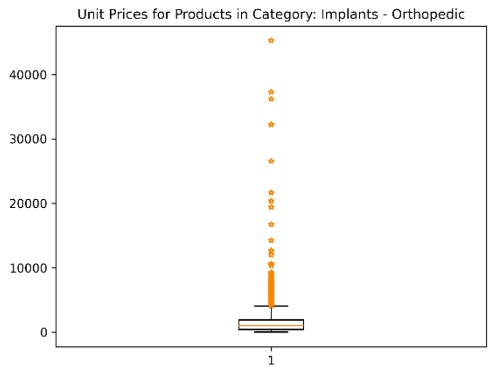

Implants - Orthopedic

This category would be considered a good example of categories that have a flatter distribution of per unit prices resulting in dissimilar rankings by quantity and money spent, thereby need for extra products.

count	2447
mean	1680
std	2631
min	3.9
25%	437
50%	1000
75%	1889
max	45280

Fig. S4. For illustration, we compare here two categories that have different share of price outliers.

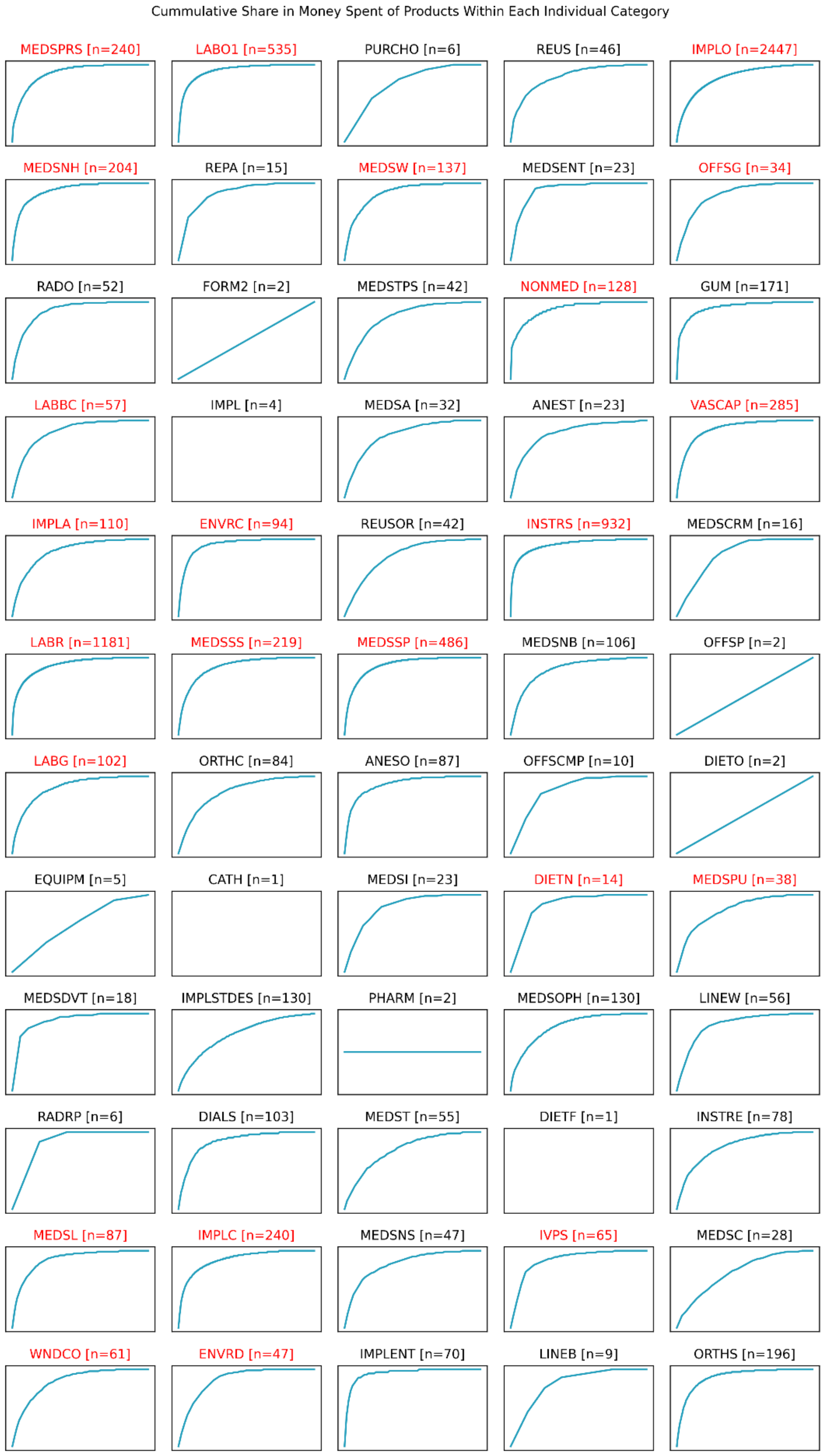

Cummulative Share in Money Spent of Products Within Each Individual Category
MEDSPRS [n=240]
LABO1 [n=535]
PURCHO [n=6]
REUS [n=46]
IMPLO [n=2447]
MEDSNH [n=204]
REPA [n=15]
MEDSW [n=137]
MEDSENT [n=23]
OFFSG [n=34]
RADO [n=52]
FORM2 [n=2]
MEDSTPS [n=42]
NONMED [n=128]
GUM [n=171]
LABBC [n=57]
IMPL [n=4]
MEDSA [n=32]
ANEST [n=23]
VASCAP [n=285]
IMPLA [n=110]
ENVRC [n=94]
REUSOR [n=42]
INSTRS [n=932]
MEDSCRM [n=16]
LABR [n=1181]
MEDSSS [n=219]
MEDSSP [n=486]
MEDSNB [n=106]
OFFSP [n=2]
LABG [n=102]
ORTHC [n=84]
ANESO [n=87]
OFFSCMP [n=10]
DIETO [n=2]
EQUIPM [n=5]
CATH [n=1]
MEDSI [n=23]
DIETN [n=14]
MEDSPU [n=38]
MEDSDVT [n=18]
IMPLSTDES [n=130]
PHARM [n=2]
MEDSOPH [n=130]
LINEW [n=56]
RADRP [n=6]
DIALS [n=103]
MEDST [n=55]
DIETF [n=1]
INSTRE [n=78]
MEDSL [n=87]
IMPLC [n=240]
MEDSNS [n=47]
IVPS [n=65]
MEDSC [n=28]
WNDCO [n=61]
ENVRD [n=47]
IMPLENT [n=70]
LINEB [n=9]
ORTHS [n=196]

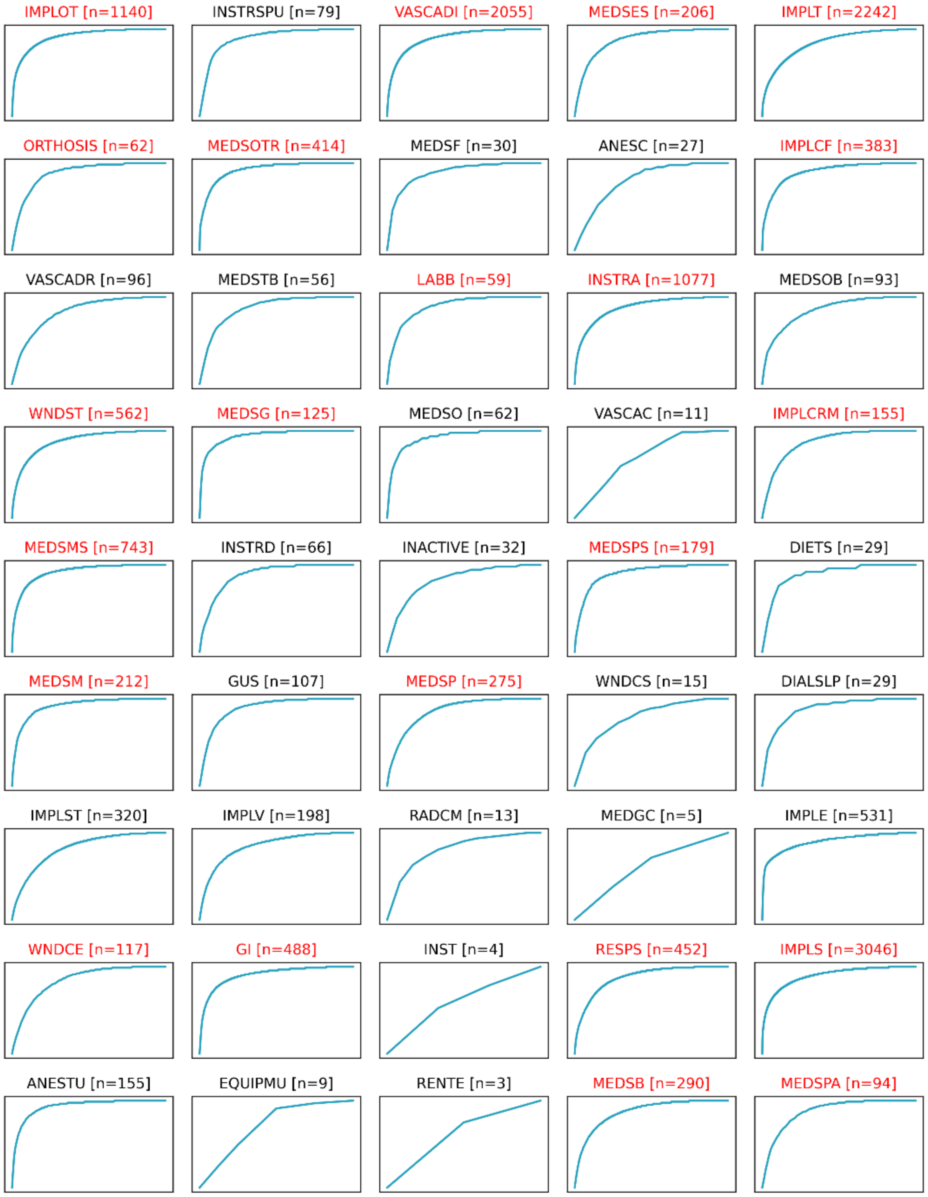

Fig S5: In this panel each chart contains the cumulative share of money received plotted over unique products sorted in descending order of quantities received for each of the 105 categories. Important to note that the scale of the x axis is not the same across all the plots. The x axis in each plot ranges from 0 to n where n is the number of unique products in each category. The charts are titled with commodity codes and number of unique products in that category. Charts with titles in red are the categories in the 45 categories selected based on money spent and quantity received criteria.

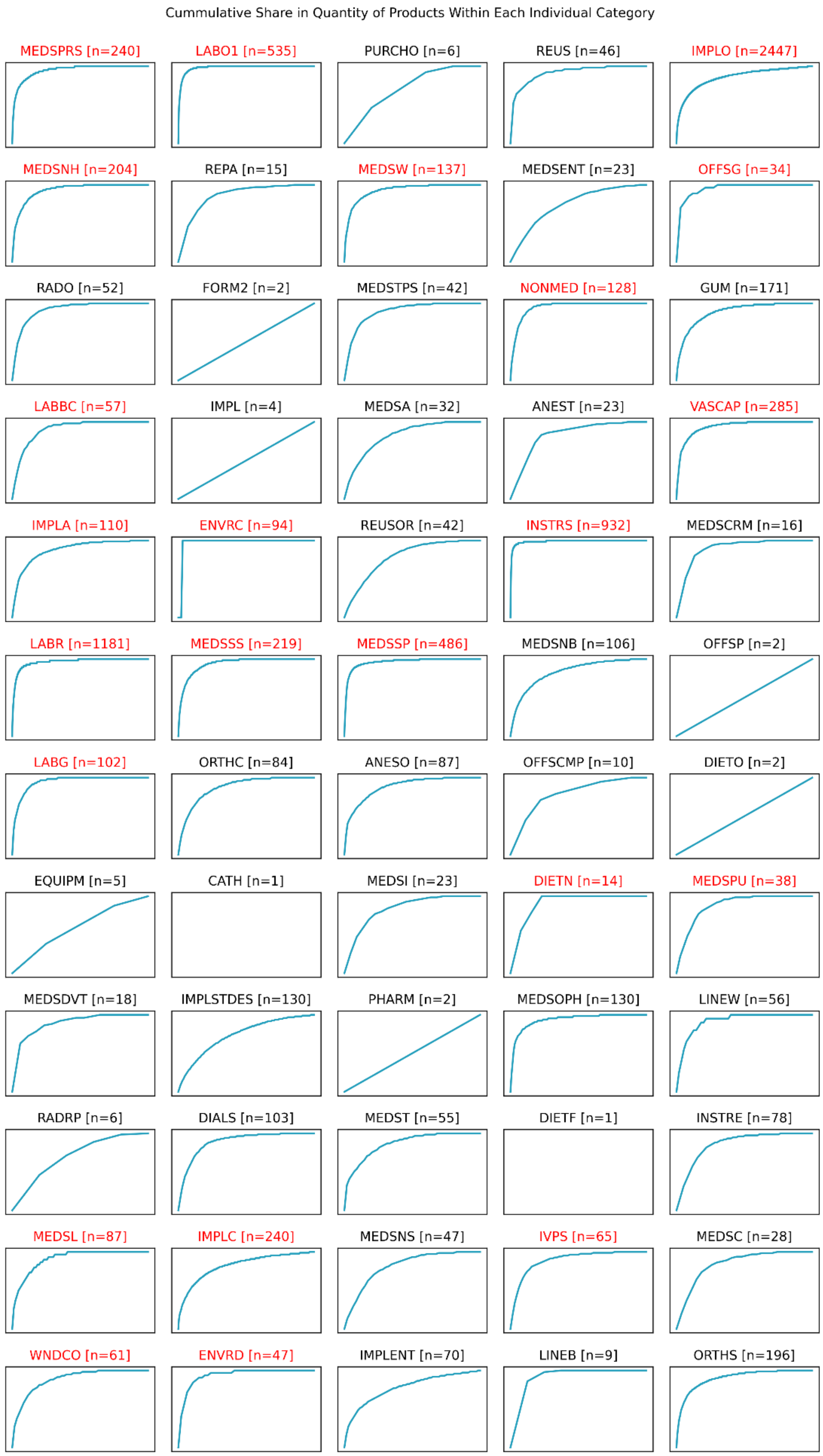
Cummulative Share in Quantity of Products Within Each Individual Category
MEDSPRS [n=240]
LABO1 [n=535]
PURCHO [n=6]
REUS [n=46]
IMPLO [n=2447]
MEDSNH [n=204]
REPA [n=15]
MEDSW [n=137]
MEDSENT [n=23]
OFFSG [n=34]
RADO [n=52]
FORM2 [n=2]
MEDSTPS [n=42]
NONMED [n=128]
GUM [n=171]
LABBC [n=57]
IMPL [n=4]
MEDSA [n=32]
ANEST [n=23]
VASCAP [n=285]
IMPLA [n=110]
ENVRC [n=94]
REUSOR [n=42]
INSTRS [n=932]
MEDSCRM [n=16]
LABR [n=1181]
MEDSSS [n=219]
MEDSSP [n=486]
MEDSNB [n=106]
OFFSP [n=2]
LABG [n=102]
ORTHC [n=84]
ANESO [n=87]
OFFSCMP [n=10]
DIETO [n=2]
EQUIPM [n=5]
CATH [n=1]
MEDSI [n=23]
DIETN [n=14]
MEDSPU [n=38]
MEDSDVT [n=18]
IMPLSTDES [n=130]
PHARM [n=2]
MEDSOPH [n=130]
LINEW [n=56]
RADRP [n=6]
DIALS [n=103]
MEDST [n=55]
DIETF [n=1]
INSTRE [n=78]
MEDSL [n=87]
IMPLC [n=240]
MEDSNS [n=47]
IVPS [n=65]
MEDSC [n=28]
WNDCO [n=61]
ENVRD [n=47]
IMPLENT [n=70]
LINEB [n=9]
ORTHS [n=196]

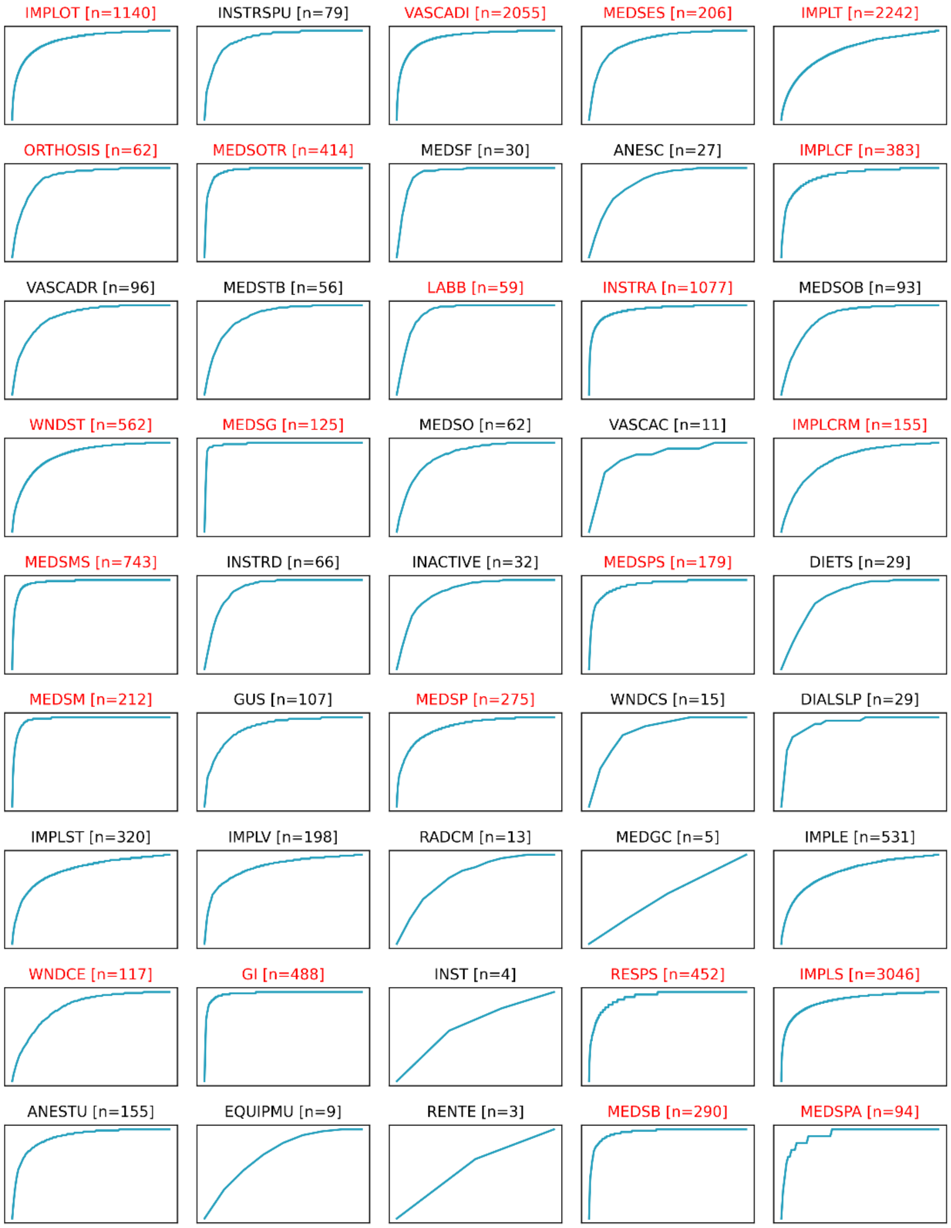

Fig S6: In this panel each chart contains the cumulative share of quantity received plotted over unique products sorted in descending order of quantities received for each of the 105 categories.

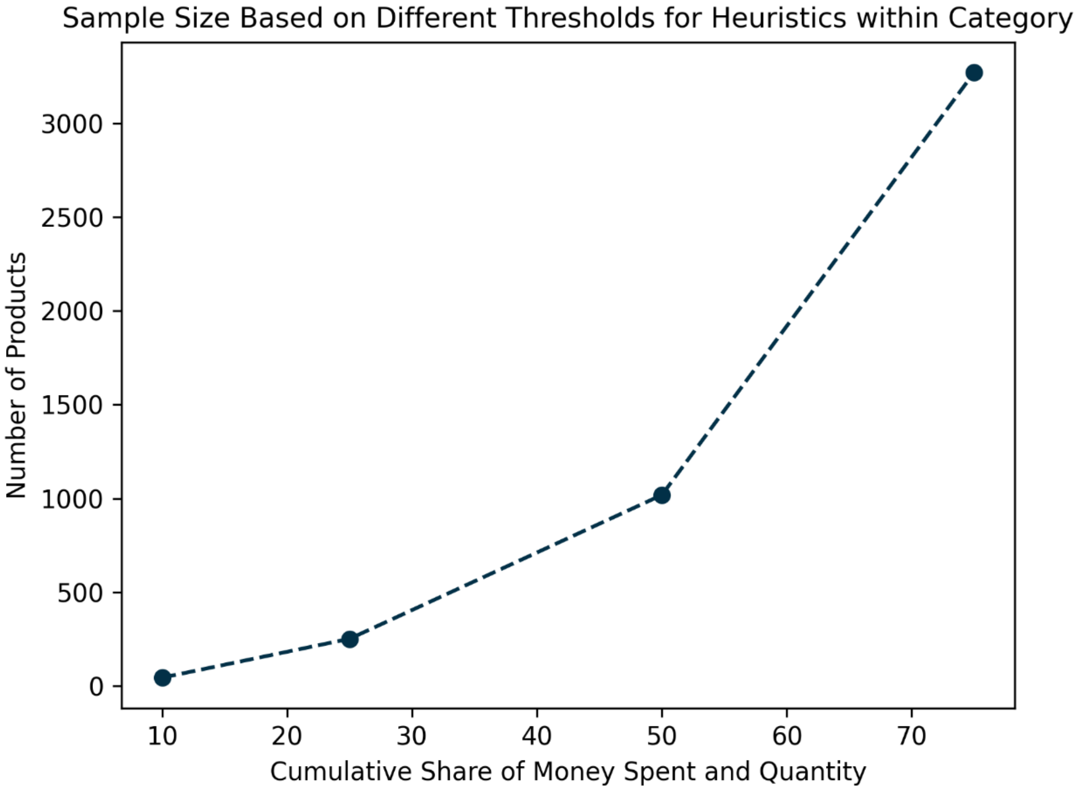

Fig S7: Total number of products in sample by applying different thresholds (10%, 25%, 50%, 75%) within category level. These thresholds apply to both the money and quantity heuristics. 50% thresholds yields ~1000 products for which data has to be collected in order to estimate lifecycle emissions.

Category	Contribution in Total Estimate	Quartiles
Med/Surg - Personal Care	17.5%	Q4
Vascular Access-Diagnostic/Intervention	8.4%	
Iv Products - Solutions	7.1%	
Med/Surg - Other Medical Supplies	6.6%	
Med/Surg - Other Surgical Supplies	6.0%	
Med/Surg - Perfusion Supplies	4.7%	
Med/Surg - Packs & Drapes	4.6%	
Med/Surg - Gloves	3.7%	
Environmental - Cleaning Supplies	3.1%	
Med/Surg - Suction/Wound Drainage	2.9%	
Implants - Other	2.3%	
Med/Surg - Needles (Hypo) & Syringes	2.1%	Q3
Lab - Blood Collection	2.0%	
Environmental - Disposable Products	2.0%	
Lab - Glassware	1.7%	
Gi (Gastrointestinal)	1.6%	
Implants - Cardiovascular	1.4%	
Med/Surg - Protective Apparel	1.3%	
Vascular Access - Peripheral Admin	1.2%	
Med/Surg - Surgery / Procedure Items	1.0%	
Med/Surg - Patient Utensils	0.9%	
Respiratory Supplies	0.6%	
Med/Surg - Bandages & Dressings	0.6%	Q2
Lab - Other	0.5%	
Orthosis Devices	0.4%	
Non-Medical Supplies	0.4%	
Med/Surg - Electrosurgical Supplies	0.3%	
Instruments - Accessories	0.3%	
Wound Closure - Endo Mechanical	0.2%	
Med/Surg - Sterilization Supplies	0.2%	
Med/Surg - Monitoring / Esu Supplies	0.2%	
Lab - Reagents	0.2%	
Lab - Bacti	0.2%	
Dietary - Non-Food	0.1%	Q1
Office Supplies - General	0.1%	
Wound Closure - Suture	0.1%	
Implants - Crm Products	0.1%	

Wound Closure - Other	0.0%	
Med/Surg - Labels	0.0%	
Implants - Orthopedic	0.0%	
Implants - Spine	0.0%	
Implants - Trauma	0.0%	
Implants - Allograft Tissue	0.0%	
Implants - Cranio-Facial	0.0%	

Table S1. Categories ordered in descending order of their share in total emissions estimated for 45 categories using bottom-up approach with money heuristic

Category	Contribution in Total Estimate	Quartiles
Med/Surg - Personal Care	17.7%	Q4
Med/Surg - Other Medical Supplies	12.6%	
Office Supplies - General	10.2%	
Iv Products - Solutions	9.3%	
Med/Surg - Protective Apparel	8.3%	
Respiratory Supplies	7.8%	
Vascular Access-Diagnostic/Intervention	5.6%	
Med/Surg - Gloves	3.8%	
Med/Surg - Suction/Wound Drainage	3.0%	
Med/Surg - Needles (Hypo) & Syringes	2.9%	
Vascular Access - Peripheral Admin	2.4%	
Environmental - Cleaning Supplies	2.0%	Q3
Environmental - Disposable Products	1.8%	
Med/Surg - Patient Utensils	1.7%	
Lab - Reagents	1.4%	
Lab - Blood Collection	1.1%	
Med/Surg - Monitoring / Esu Supplies	0.9%	
Med/Surg - Packs & Drapes	0.7%	
Wound Closure - Endo Mechanical	0.6%	
Med/Surg - Bandages & Dressings	0.6%	
Med/Surg - Sterilization Supplies	0.5%	
Lab - Glassware	0.5%	
Gi (Gastrointestinal)	0.5%	Q2
Med/Surg - Labels	0.5%	
Lab - Bacti	0.5%	
Med/Surg - Other Surgical Supplies	0.5%	
Lab - Other	0.3%	
Med/Surg - Surgery / Procedure Items	0.3%	
Orthosis Devices	0.2%	
Med/Surg - Perfusion Supplies	0.2%	
Dietary - Non-Food	0.2%	
Wound Closure - Suture	0.1%	
Med/Surg - Electrosurgical Supplies	0.1%	
Implants - Other	0.0%	Q1
Instruments - Accessories	0.0%	
Wound Closure - Other	0.0%	
Implants - Cardiovascular	0.0%	
Implants - Spine	0.0%	

Non-Medical Supplies	0.0%	
Implants - Orthopedic	0.0%	
Implants - Trauma	0.0%	
Implants - Allograft Tissue	0.0%	
Implants - Crm Products	0.0%	
Implants - Cranio-Facial	0.0%	

Table S2. Categories ordered in descending order of their share in total emissions estimated for 45 categories using bottom-up approach with quantity heuristic

Category	Contribution in Total Estimate	Quartiles
Implants - Spine	11.19%	Q4
Lab - Reagents	9.83%	
Implants - Other	8.08%	
Vascular Access-Diagnostic/Intervention	6.67%	
Implants - Orthopedic	4.66%	
Implants - Cardiovascular	3.53%	
Med/Surg - Packs & Drapes	3.15%	
Med/Surg - Other Surgical Supplies	2.94%	
Iv Products - Solutions	2.73%	
Vascular Access - Peripheral Admin	2.57%	
Med/Surg - Personal Care	2.36%	
Med/Surg - Other Medical Supplies	2.21%	Q3
Implants - Crm Products	2.11%	
Med/Surg - Monitoring / Esu Supplies	1.96%	
Med/Surg - Electrosurgical Supplies	1.87%	
Orthosis Devices	1.76%	
Med/Surg - Surgery / Procedure Items	1.48%	
Wound Closure - Suture	1.33%	
Gi (Gastrointestinal)	1.27%	
Med/Surg - Gloves	1.21%	
Instruments - Accessories	1.14%	
Implants - Allograft Tissue	1.13%	
Respiratory Supplies	1.10%	Q2
Implants - Trauma	1.08%	
Wound Closure - Endo Mechanical	1.05%	
Wound Closure - Other	1.01%	
Med/Surg - Perfusion Supplies	1.01%	
Implants - Cranio-Facial	1.00%	
Environmental - Disposable Products	0.93%	
Med/Surg - Bandages & Dressings	0.89%	
Implants - Stents - Non Des	0.88%	
Implants - Vascular	0.76%	
Implants - Ear-Nose-Throat	0.74%	
Med/Surg - Sterilization Supplies	0.72%	Q1
Instruments - Surgical	0.70%	
Med/Surg - Suction/Wound Drainage	0.69%	
Radiology - Radioactive Products	0.62%	
Med/Surg - Protective Apparel	0.62%	

Med/Surg - Needles (Hypo) & Syringes	0.61%	
Environmental - Cleaning Supplies	0.56%	
Implants - Eye	0.55%	
Gu (Genito-Urinary) - Medical	0.53%	
Dialysis Supplies	0.53%	
Anesthesia Supplies - Tubes	0.42%	

Table S3. Categories ordered in descending order of their share in total emissions estimated for 45 categories using top-down approach with Engie tool.

Category	Number of Optimum Clusters
Implants - Spine	526
Implants - Orthopedic	507
Vascular Access-Diagnostic/Intervention	406
Implants - Trauma	371
Instruments - Accessories	310
Implants - Other	214
Instruments - Surgical	187
Lab - Other	101
Gi (Gastrointestinal)	88
Implants - Stents - Non Des	76
Med/Surg - Surgery / Procedure Items	72
Implants - Eye	69
Implants - Vascular	63
Med/Surg - Other Medical Supplies	62
Med/Surg - Personal Care	52
Implants - Cranio-Facial	49
Vascular Access - Peripheral Admin	33
Wound Closure - Suture	32
Implants - Allograft Tissue	28
Med/Surg - Perfusion Supplies	25
Med/Surg - Gloves	23
Med/Surg - Sterilization Supplies	18
Med/Surg - Needles (Spinal)	16
Wound Closure - Endo Mechanical	13
Implants - Stents - Des	11
Dialysis Supplies	10
Orthopedic - Casting	9
Inactive Items	6
Linen - Wearing Apparel	6
Dietary - Formula / Supplements	4
Vascular Access-Coils	4
Wound Closure - Other	4
Gu (Genito-Urinary) - Medical	3
Rentals - Equipment	3
Repair Parts	3
Dietary - Non-Food	2
Dietary - Outside Purchases	2
Environmental - Cleaning Supplies	2
Forms	2
Implants - Crm Products	2
Implants - Ear-Nose-Throat	2
Instruments - Single Patient Use	2
Med/Surg - Labels	2
Med/Surg - Ostomy Supplies	2
Med/Surg - Suction/Wound Drainage	2
Office Supplies - Paper	2
Pharaceuticals	2
Reusable Supplies	2

Anesthesia Supplies - Circuits	1
Anesthesia Supplies - Other	1
Anesthesia Supplies - Trays	1
Anesthesia Supplies - Tubes	1
Catheter-Misc	1
Dialysis/Pheresis	1
Dietary - Food / Drinks	1
Environmental - Disposable Products	1
Equipment - Other Minor < 200	1
Equipment -Other Minor > 200	1
Gu (Genito-Urinary) - Surgical	1
Implants - Cardiovascular	1
Implants And Prosthesis	1
Instruments	1
Instruments - Diagnostic	1
Instruments - Endo	1
Iv Products - Solutions	1
Lab - Bacti	1
Lab - Blood Collection	1
Lab - Glassware	1
Lab - Reagents	1
Linen - Bedding / Towels	1
Med/Surg - Adapters	1
Med/Surg - Bandages & Dressings	1
Med/Surg - Connectors	1
Med/Surg - Crm (Non-Implant)	1
Med/Surg - Dvt/Scd Supplies	1
Med/Surg - Ear-Nose-Throat	1
Med/Surg - Electrosurgical Supplies	1
Med/Surg - Filters	1
Med/Surg - Irrigation	1
Med/Surg - Monitoring / Esu Supplies	1
Med/Surg - Needles (Biopsy)	1
Med/Surg - Needles (Hypo) & Syringes	1
Med/Surg - Ob/Gyn Supplies	1
Med/Surg - Ophthalmology	1
Med/Surg - Other Surgical Supplies	1
Med/Surg - Packs & Drapes	1
Med/Surg - Patient Utensils	1
Med/Surg - Procedure Trays	1
Med/Surg - Protective Apparel	1
Med/Surg - Tapes	1
Med/Surg - Tubing	1
Medical Gases - Cylinder	1
Non-Medical Supplies	1
Office Supplies - Computer	1
Office Supplies - General	1
Orthopedic - Softgoods	1
Orthosis Devices	1

Purchased Services - Other	1
Radiology - Contrast Media	1
Radiology - Other	1
Radiology - Radioactive Products	1
Respiratory Supplies	1
Reusable Supplies - Or	1
Vascular Access - Drug Delivery	1
Wound Closure - Skin External	1

Table S4. Categories ordered in descending order by optimum number of clusters as per our machine learning based analysis of product text descriptions.

UCSF Hospital Data - Product Categories	Engie Categories
LAB - BACTI	Ambulatory health care services - Medical laboratories
LAB - BLOOD COLLECTION	Ambulatory health care services - Medical laboratories
LAB - GLASSWARE	Ambulatory health care services - Medical laboratories
LAB - OTHER	Ambulatory health care services - Medical laboratories
LINEN - WEARING APPAREL	Apparel and leather and allied products - Clothing
MED/SURG - TAPES	Chemical products - Adhesives
LAB - REAGENTS	Chemical products - Chemicals (except basic chemicals, agrichemicals, polymers, paints, pharmaceuticals, soaps, cleaning compounds)
MEDICAL GASES - CYLINDER	Chemical products - Compressed Gases
IV PRODUCTS - SOLUTIONS	Chemical products - Other basic inorganic chemicals
RADIOLOGY - CONTRAST MEDIA	Chemical products - Other basic organic chemicals
PHARACEUTICALS	Chemical products - Pharmaceutical products (pills, powders, solutions, etc.)
ENVIRONMENTAL - CLEANING SUPPLIES	Chemical products - Soap and cleaning compounds
MED/SURG - PERSONAL CARE	Chemical products - Toiletries
OFFICE SUPPLIES - COMPUTER	Computer and electronic products - Computers
MED/SURG - CRM (NON-IMPLANT)	Computer and electronic products - Electromedical apparatuses
EQUIPMENT - OTHER MINOR < 200	Computer and electronic products - General
EQUIPMENT -OTHER MINOR > 200	Computer and electronic products - General
DIETARY - FOOD / DRINKS	Food Manufacturing - General
DIETARY - FORMULA / SUPPLEMENTS	Food Manufacturing - General
NON-MEDICAL SUPPLIES	Miscellaneous manufacturing - General
DIETARY - OUTSIDE PURCHASES	Miscellaneous manufacturing - General
MED/SURG - PATIENT UTENSILS	Miscellaneous manufacturing - General
OFFICE SUPPLIES - GENERAL	Miscellaneous manufacturing - Office supplies (not paper)
CATHETER-MISC	Miscellaneous manufacturing - Surgical and medical instruments
INSTRUMENTS	Miscellaneous manufacturing - Surgical and medical instruments
INSTRUMENTS - ACCESSORIES	Miscellaneous manufacturing - Surgical and medical instruments
INSTRUMENTS - DIAGNOSTIC	Miscellaneous manufacturing - Surgical and medical instruments
INSTRUMENTS - ENDO	Miscellaneous manufacturing - Surgical and medical instruments
INSTRUMENTS - SINGLE PATIENT USE	Miscellaneous manufacturing - Surgical and medical instruments
INSTRUMENTS - SURGICAL	Miscellaneous manufacturing - Surgical and medical instruments
MED/SURG - NEEDLES (BIOPSY)	Miscellaneous manufacturing - Surgical and medical instruments
MED/SURG - NEEDLES (HYPO) & SYRINGES	Miscellaneous manufacturing - Surgical and medical instruments
MED/SURG - NEEDLES (SPINAL)	Miscellaneous manufacturing - Surgical and medical instruments
MED/SURG - ELECTROSURGICAL SUPPLIES	Miscellaneous manufacturing - Surgical appliance and supplies
MED/SURG - MONITORING / ESU SUPPLIES	Miscellaneous manufacturing - Surgical appliance and supplies
ANESTHESIA SUPPLIES - CIRCUITS	Miscellaneous manufacturing - Surgical appliance and supplies
RADIOLOGY - OTHER	Miscellaneous manufacturing - Surgical appliance and supplies
RADIOLOGY - RADIOACTIVE PRODUCTS	Miscellaneous manufacturing - Surgical appliance and supplies

ANESTHESIA SUPPLIES - OTHER	Miscellaneous manufacturing - Surgical appliance and supplies
ANESTHESIA SUPPLIES - TRAYS	Miscellaneous manufacturing - Surgical appliance and supplies
ANESTHESIA SUPPLIES - TUBES	Miscellaneous manufacturing - Surgical appliance and supplies
DIALYSIS SUPPLIES	Miscellaneous manufacturing - Surgical appliance and supplies
MED/SURG - BANDAGES & DRESSINGS	Miscellaneous manufacturing - Surgical appliance and supplies
MED/SURG - DVT/SCD SUPPLIES	Miscellaneous manufacturing - Surgical appliance and supplies
MED/SURG - GLOVES	Miscellaneous manufacturing - Surgical appliance and supplies
MED/SURG - OB/GYN SUPPLIES	Miscellaneous manufacturing - Surgical appliance and supplies
MED/SURG - OSTOMY SUPPLIES	Miscellaneous manufacturing - Surgical appliance and supplies
MED/SURG - OTHER MEDICAL SUPPLIES	Miscellaneous manufacturing - Surgical appliance and supplies
MED/SURG - OTHER SURGICAL SUPPLIES	Miscellaneous manufacturing - Surgical appliance and supplies
MED/SURG - PERFUSION SUPPLIES	Miscellaneous manufacturing - Surgical appliance and supplies
MED/SURG - PROCEDURE TRAYS	Miscellaneous manufacturing - Surgical appliance and supplies
MED/SURG - STERILIZATION SUPPLIES	Miscellaneous manufacturing - Surgical appliance and supplies
MED/SURG - SUCTION/WOUND DRAINAGE	Miscellaneous manufacturing - Surgical appliance and supplies
MED/SURG - SURGERY / PROCEDURE ITEMS	Miscellaneous manufacturing - Surgical appliance and supplies
MED/SURG - TUBING	Miscellaneous manufacturing - Surgical appliance and supplies
RESPIRATORY SUPPLIES	Miscellaneous manufacturing - Surgical appliance and supplies
REUSABLE SUPPLIES	Miscellaneous manufacturing - Surgical appliance and supplies
REUSABLE SUPPLIES - OR	Miscellaneous manufacturing - Surgical appliance and supplies
MED/SURG - PROTECTIVE APPAREL	Miscellaneous manufacturing - Surgical appliance and supplies
WOUND CLOSURE - ENDO MECHANICAL	Miscellaneous manufacturing - Surgical appliance and supplies
WOUND CLOSURE - OTHER	Miscellaneous manufacturing - Surgical appliance and supplies
WOUND CLOSURE - SKIN EXTERNAL	Miscellaneous manufacturing - Surgical appliance and supplies
WOUND CLOSURE - SUTURE	Miscellaneous manufacturing - Surgical appliance and supplies
MED/SURG - ADAPTERS	Miscellaneous manufacturing - Surgical appliance and supplies
MED/SURG - CONNECTORS	Miscellaneous manufacturing - Surgical appliance and supplies
MED/SURG - FILTERS	Miscellaneous manufacturing - Surgical appliance and supplies
MED/SURG - IRRIGATION	Miscellaneous manufacturing - Surgical appliance and supplies
IMPLANTS - ALLOGRAFT TISSUE	Miscellaneous manufacturing - Surgical appliance and supplies
IMPLANTS - CARDIOVASCULAR	Miscellaneous manufacturing - Surgical appliance and supplies
IMPLANTS - CRANIO-FACIAL	Miscellaneous manufacturing - Surgical appliance and supplies
IMPLANTS - CRM PRODUCTS	Miscellaneous manufacturing - Surgical appliance and supplies
IMPLANTS - EAR-NOSE-THROAT	Miscellaneous manufacturing - Surgical appliance and supplies
IMPLANTS - EYE	Miscellaneous manufacturing - Surgical appliance and supplies
IMPLANTS - ORTHOPEDIC	Miscellaneous manufacturing - Surgical appliance and supplies
IMPLANTS - OTHER	Miscellaneous manufacturing - Surgical appliance and supplies
IMPLANTS - SPINE	Miscellaneous manufacturing - Surgical appliance and supplies
IMPLANTS - STENTS - DES	Miscellaneous manufacturing - Surgical appliance and supplies
IMPLANTS - STENTS - NON DES	Miscellaneous manufacturing - Surgical appliance and supplies
IMPLANTS - TRAUMA	Miscellaneous manufacturing - Surgical appliance and supplies
IMPLANTS - VASCULAR	Miscellaneous manufacturing - Surgical appliance and supplies

IMPLANTS AND PROSTHESIS	Miscellaneous manufacturing - Surgical appliance and supplies
DIALYSIS/PHERESIS	Miscellaneous manufacturing - Surgical appliance and supplies
GI (GASTROINTESTINAL)	Miscellaneous manufacturing - Surgical appliance and supplies
GU (GENITO-URINARY) - MEDICAL	Miscellaneous manufacturing - Surgical appliance and supplies
GU (GENITO-URINARY) - SURGICAL	Miscellaneous manufacturing - Surgical appliance and supplies
INACTIVE ITEMS	Miscellaneous manufacturing - Surgical appliance and supplies
MED/SURG - EAR-NOSE-THROAT	Miscellaneous manufacturing - Surgical appliance and supplies
MED/SURG - OPHTHALMOLOGY	Miscellaneous manufacturing - Surgical appliance and supplies
ORTHOPEDIC - CASTING	Miscellaneous manufacturing - Surgical appliance and supplies
ORTHOPEDIC - SOFTGOODS	Miscellaneous manufacturing - Surgical appliance and supplies
ORTHOSIS DEVICES	Miscellaneous manufacturing - Surgical appliance and supplies
VASCULAR ACCESS - DRUG DELIVERY	Miscellaneous manufacturing - Surgical appliance and supplies
VASCULAR ACCESS - PERIPHERAL ADMIN	Miscellaneous manufacturing - Surgical appliance and supplies
VASCULAR ACCESS-COILS	Miscellaneous manufacturing - Surgical appliance and supplies
VASCULAR ACCESS-DIAGNOSTIC/INTERVENTION	Miscellaneous manufacturing - Surgical appliance and supplies
MED/SURG - PACKS & DRAPES	Miscellaneous manufacturing - Surgical appliance and supplies
REPAIR PARTS	Other services, except government - Commercial machinery repair
RENTALS - EQUIPMENT	Other services, except government - General
PURCHASED SERVICES - OTHER	Other services, except government - General
DIETARY - NON-FOOD	Paper products - All other converted paper products
MED/SURG - LABELS	Paper products - General
OFFICE SUPPLIES - PAPER	Paper products - Paper
ENVIRONMENTAL - DISPOSABLE PRODUCTS	Paper products - Sanitary paper (tissues, napkins, diapers, etc.)
LINEN - BEDDING / TOWELS	Textile mills and textile product mills - Curtains and linens
FORMS	Paper products - Paper

Table S5. 105 categories in the Hospital data are matched to appropriate categories in the Engie tool for calculating the top-down EEIO-based emissions estimate.

SI References

Hamerly, G., & Elkan, C. (2004). Learning the k in k-means. *Advances in Neural Information Processing System 16 (NIPS 2003)*, *16*, 281. https://proceedings.neurips.cc/paper_files/paper/2003/file/234833147b97bb6aed53a8f4f1c7a7d8-Paper.pdf

Mikolov, T., Chen, K., Corrado, G., & Dean, J. (2013). *Efficient Estimation of Word Representations in Vector Space* (arXiv:1301.3781). arXiv. http://arxiv.org/abs/1301.3781
